# Coffee Husk-Derived Activated Carbon for the Adsorptive Removal of Imatinib Mesylate from Water: Kinetic, Isotherm, Thermodynamic, and DFT Studies

**DOI:** 10.3390/molecules31193391

**Published:** 2026-09-24

**Authors:** Vivian Párraga-García, Jeniffer Chiquito, Carolina Montero-Calderón, Lucia E. Manangon-Perugachi, Alejandro Altamirano-Briones, Erika Góngora-Muñoz, Joan Manuel Rodríguez-Díaz, Michael Macías-Pro, Santiago Guerrero-Jaramillo, Thibault Terencio, Pablo A. Cisneros-Pérez, Luis Corredor-González, Patricio J. Espinoza-Montero

**Affiliations:** 1 Carrera de Ingeniería Química, Facultad de Ingeniería y Ciencias Aplicadas, Universidad Técnica de Manabí (UTM), Portoviejo 130105, Ecuador; vivilipg02@gmail.com (V.P.-G.); jchiquito9471@utm.edu.ec (J.C.); 2 Facultad de Ciencias Exactas Naturales y Ambientales, Pontificia Universidad Católica del Ecuador, Quito 170525, Ecuador; sfguerreroj@puce.edu.ec; 3 Facultad de Ingeniería Química, Universidad Central del Ecuador, Quito 170402, Ecuador; cdmontero@uce.edu.ec; 4 Department of Extractive Metallurgy, Escuela Politécnica Nacional, Quito 170525, Ecuador; lucia.manangon@epn.edu.ec; 5 Carrera de Agroindustria, Escuela Superior Politécnica Agropecuaria de Manabí “Manuel Félix López”, Calceta 130250, Ecuador; galtamirano@espam.edu.ec; 6 Departamento de Química, Facultad de Ciencias Básicas, Universidad Técnica de Manabí (UTM), Portoviejo 130105, Ecuador; erika.gongora@utm.edu.ec; 7 Departamento de Procesos Químicos, Biotecnología y Alimentos, Facultad de Ingenierías y Ciencias Aplicadas, Universidad Técnica de Manabí, Portoviejo 130105, Ecuador; joan.rodriguez@utm.edu.ec; 8 Research Group Agroecosystems Functioning Climate Change—FAGROCLIM, Faculty of Agriculture Engineering, Universidad Técnica de Manabí (UTM), Lodana 13132, Ecuador; michael.macias@utm.edu.ec; 9 School of Chemistry and Engineering, Yachay Tech University, Urcuqui 100119, Ecuador; tthibault@yachaytech.edu.ec (T.T.); pcisneros@yachaytech.edu.ec (P.A.C.-P.); lcorredor@yachaytech.edu.ec (L.C.-G.)

**Keywords:** coffee husk waste, imatinib mesylate, activated carbon, sustainable water treatment

## Abstract

Coffee husk-derived activated carbon prepared by H_3_PO_4_ chemical activation was investigated for the removal of imatinib mesylate from aqueous solutions. Structural and chemical characterization using N_2_ physisorption, SEM, FTIR, Raman spectroscopy, and thermogravimetric analysis revealed a porous carbon matrix containing oxygenated functional groups and a defective turbostratic structure. The adsorption behavior was evaluated through batch experiments by examining the effects of pH, adsorbent dosage, particle size, contact time, and initial drug concentration. Under optimized conditions (300 µm particle size, 0.6 g adsorbent dosage, and pH 6), imatinib mesylate removal reached 97.9%, with equilibrium achieved within 60 min. The adsorption kinetics followed the pseudo-second-order model (R^2^ = 0.946–0.984), while equilibrium data were best described by the Sips isotherm model (R^2^ > 0.99), indicating adsorption on a heterogeneous surface. Density functional theory calculations confirmed the contribution of oxygen-containing groups, with carboxyl-functionalized carbon exhibiting the strongest interaction with imatinib mesylate (−105.3 kJ mol^−1^). These findings highlight the potential of coffee husk-derived activated carbon as a sustainable adsorbent for pharmaceutical contaminant removal.

## 1. Introduction

The increasing demand for water, driven by population growth and industrial development, together with the progressive deterioration of water quality, has made the shortage of potable water a critical global challenge [1]. Among the numerous factors affecting water quality, the occurrence of cytotoxic pharmaceuticals has become a growing concern. Imatinib mesylate (IM) has attracted attention because of its persistence, potential toxicological effects, and resistance to conventional wastewater treatment processes [2]. IM is widely used in advanced cancer therapies, including the treatment of chronic myeloid leukaemia and gastrointestinal stromal tumours [3]. However, up to 81% of the administered dose may be excreted unchanged or as active metabolites, enabling its entry into wastewater streams and natural aquatic systems, where it has been detected at concentrations in the nanogram-per-litre range [4]. The persistence of IM in aquatic environments poses a significant environmental challenge, as conventional wastewater treatment plants are often unable to achieve complete removal of such compounds. This highlights the need for efficient and sustainable treatment technologies capable of protecting water quality and environmental integrity [5].

Conventional wastewater treatment technologies, including biological processes and chlorination, present important limitations for the removal of pharmaceutical contaminants, particularly cytostatic agents such as IM [6]. These processes are frequently insufficient for eliminating structurally complex molecules at trace concentrations and, in some cases, may promote the formation of transformation by-products with unknown or potentially higher toxicity than the parent compound [7]. Although advanced oxidation processes have been widely investigated for pharmaceutical degradation, their practical application remains limited by incomplete mineralization, high operational costs, and the possible formation of intermediate metabolites [8]. In this context, adsorption has emerged as a promising alternative because it can efficiently retain target molecules from aqueous media without necessarily generating secondary transformation products [7].

A broad range of adsorbent materials has been investigated for the removal of pharmaceutical contaminants, including commercial activated carbons, carbon nanotubes, biochars, and functionalized composites. However, information regarding the efficient removal of cytostatic drugs using activated carbons derived from residual biomass remains limited. This gap is particularly relevant given the need for low-cost, sustainable, and high-performance materials capable of mitigating contamination by active anticancer compounds in wastewater. Therefore, the development of biomass-derived activated carbons represents an attractive strategy that combines environmental remediation with the valorisation of agro-industrial residues.

The use of agricultural waste as a precursor for sustainable adsorbents has received increasing attention because of its economic feasibility, wide availability, and environmental benefits [9]. Among these residues, coffee husk is a particularly promising precursor for activated carbon production due to its abundance in coffee-producing regions, especially in Latin America [10]. Its lignocellulosic composition, rich in cellulose, hemicellulose, and lignin, favours the development of activated carbons with well-defined micro- and mesoporous structures, as well as surfaces enriched with oxygen-containing functional groups [11]. These textural and chemical properties are essential for promoting high adsorption affinity toward complex organic pollutants, including cytostatic drugs [12]. Previous studies, such as that reported by Mustafa et al. [13], have demonstrated the removal of pazopanib using conventionally activated carbon, attributing its adsorption performance to a large surface area and a well-developed microporous framework. Nevertheless, studies focused on activated carbon derived specifically from coffee husks for the removal of persistent pharmaceutical contaminants, particularly IM, remain scarce. Addressing this limitation is important for advancing sustainable and resource-efficient technologies for water remediation.

In this context, the present study evaluates the adsorption performance of activated carbon produced from coffee husks for the removal of imatinib mesylate from aqueous solutions. The adsorption process is investigated through kinetic, equilibrium, and thermodynamic analyses to determine the operating conditions that maximize removal efficiency and to elucidate the mechanisms governing IM uptake. This work contributes to the development of sustainable biomass-derived adsorbents while providing insight into the potential application of coffee husk activated carbon for the treatment of pharmaceutical-contaminated water.

## 2. Results and Discussion

### 2.1. Characterization of Activated Carbon

After H_3_PO_4_ chemical activation and subsequent thermal treatment, the coffee husk-derived activated carbon (AC) exhibited a Brunauer–Emmett–Teller (BET) surface area of 565 m^2^ g^−1^, a total pore volume of 0.509 cm^3^ g^−1^, and a micropore volume of 0.209 cm^3^ g^−1^ at 77 K. The PSD shows a predominant microporous contribution, with the main pore width centered at ≈1.2 nm, along with a broader contribution extending into the mesoporous range. The coexistence of pores in these different size ranges further supports the hierarchical porous structure of the AC. This hierarchical pore distribution is advantageous for adsorption because micropores provide abundant adsorption sites, whereas larger pores facilitate molecular diffusion and mass transfer. These results are consistent with those reported by Fatma et al. [14], who obtained a surface area of 583 m^2^ g^−1^ for activated carbon prepared from corn stigmata under comparable activation conditions. Overall, the observed textural characteristics highlight the effectiveness of phosphoric acid as a chemical activating agent, promoting hydrolysis, dehydration, and depolymerization of the lignocellulosic matrix and thereby generating an extensive porous network.

The FTIR spectra of CH and AC are shown in Figure 1. The spectrum of raw CH displayed a broad absorption band centred at approximately 3250 cm^−1^, assigned to O–H stretching vibrations in hydroxyl-rich polysaccharides and polyphenols typical of lignocellulosic biomass. The broadness of this band indicates extensive hydrogen bonding among cellulose, hemicellulose, lignin, and residual moisture. A shoulder near 2920 cm^−1^ was associated with aliphatic C–H stretching vibrations of –CH_2_ and –CH_3_ groups present in the side chains of these biopolymers [15]. In the region below 1800 cm^−1^, characteristic bands of lignocellulosic materials were observed, including a carbonyl band at approximately 1730 cm^−1^, attributed to C=O stretching in esters and carboxylic acids from hemicellulose. Aromatic C=C vibrations between 1600 and 1510 cm^−1^ were related to lignin, whereas the intense C–O stretching envelope at approximately 1030 cm^−1^ was assigned to secondary alcohols and ether linkages in cellulose [16].

After phosphoric acid activation and thermal treatment, the FTIR spectrum of AC became markedly less intense and feature-poor, indicating extensive structural transformation of the precursor. The disappearance or strong attenuation of the bands at 3250 and 2920 cm^−1^ confirmed dehydroxylation and dehydrogenation processes associated with dehydration, decarboxylation, and cleavage of aliphatic chains during thermal treatment [17]. Similarly, the attenuation of the carbonyl band at 1730 cm^−1^ and the reduced intensity of the band at 1030 cm^−1^ evidenced the degradation of hemicellulose and cellulose, consistent with mass loss and pore development during chemical activation [18]. A residual broad band centred at approximately 1570 cm^−1^ was attributed to graphitic C=C stretching in polyaromatic domains, suggesting the formation of an aromatized carbon matrix. This structural feature is relevant for adsorption because aromatic domains can favour π–π interactions with pharmaceutical molecules containing conjugated structures [19].

The absorption bands observed in the 1200–900 cm^−1^ region were attributed to oxygen-containing surface functional groups, including phenolic and hydroxyl moieties, as well as phosphorus-containing functionalities generated during chemical activation. In particular, the appearance of shoulder bands in this region may be associated with P–O–C and P–O–P linkages formed through cross-linking reactions between H_3_PO_4_ and the carbon framework [20]. These oxygenated and phosphorus-containing surface species may act as Lewis acid sites and contribute to electrostatic interactions and hydrogen-bond formation with the ionizable functional groups of imatinib mesylate, thereby enhancing adsorption affinity.

The FTIR results demonstrate the transformation of the oxygen-rich lignocellulosic precursor into a more aromatized carbonaceous structure containing surface heteroatoms. This combination of aromatic domains, residual oxygenated groups, and phosphorus-containing functionalities confirms the successful synthesis of a porous adsorbent with chemical features favourable for the adsorption of cytostatic drugs such as imatinib mesylate.

The Raman spectrum of the coffee husk-derived activated carbon is presented in Figure 2. The spectrum exhibited the characteristic D and G bands of disordered carbon materials, centred at 1338 and 1587 cm^−1^, respectively [21]. The G band, corresponding to the E_2g_ vibrational mode, is associated with the in-plane stretching of sp^2^-bonded carbon atoms in aromatic domains [22]. In contrast, the D band is related to the breathing mode of sp^2^ carbon rings and becomes active in the presence of structural defects, including edges, vacancies, and finite crystallite dimensions. The relatively high intensity and broadening of the D band indicate a high defect density and the presence of small aromatic domains.

Additional contributions were observed in the Raman spectrum. The D3 band at approximately 1425 cm^−1^ suggests the presence of amorphous and highly disordered sp^2^ carbon, whereas the D4 band at approximately 1208 cm^−1^ is commonly attributed to sp^2^–sp^3^ disordered structures and oxygen-containing functional groups in chemically activated carbons. The broad character of the D and D3 bands further supports the formation of a turbostratic carbon arrangement with limited graphitic ordering [23,24]. Therefore, Raman analysis confirms that the material consists of small sp^2^ aromatic clusters embedded in a highly defective and partially amorphous carbon matrix, which is consistent with the structural characteristics expected for chemically activated porous carbons [25].

The thermal stability of the activated carbon was evaluated by thermogravimetric analysis, as shown in Figure 3. The TGA profile displayed three main mass-loss regions, corresponding to the sequential removal of physically adsorbed species, decomposition of surface oxygen functionalities, and degradation of the carbonaceous framework. The first mass-loss region, occurring from room temperature to approximately 473 K, was attributed to the evaporation of physically adsorbed water and low-molecular-weight volatile compounds. In this stage, the sample showed an abrupt mass loss of approximately 20 wt%, indicating a relatively high moisture and volatile content. This behaviour is consistent with the porous structure and high specific surface area of the activated carbon, which favour water retention within the pore network [26].

The second stage, extending from approximately 473 to 873 K, was characterized by a substantial decrease in mass from about 75 to 25 wt%. This mass loss is mainly associated with the thermal decomposition of oxygen-containing surface functional groups, including carboxylic acids, anhydrides, lactones, phenolic hydroxyls, and carbonyl groups. The magnitude of this degradation stage suggests a relatively high density of oxygenated functionalities on the AC surface. These groups are important for adsorption because they influence surface polarity, acid–base properties, and the availability of active sites for interactions with imatinib mesylate [27]. Above approximately 673 K, the degradation of surface functionalities increasingly overlapped with the oxidative decomposition of the carbonaceous matrix, leading to progressive combustion of the carbon skeleton up to 973 K.

At the end of the analysis, approximately 18 wt% of the initial mass remained as residue. This residual fraction can be attributed to inorganic ash derived from mineral constituents naturally present in the coffee husk precursor or introduced during the activation process. The TGA results therefore support the presence of a porous carbon matrix containing thermally labile oxygenated surface groups and a measurable inorganic fraction, both of which may influence the adsorption behaviour of the material.

The thermal treatment under static air was employed as part of an H_3_PO_4_-assisted chemical activation procedure. In this process, phosphoric acid acts as a dehydrating and cross-linking agent, promoting bond cleavage, condensation, aromatization, and stabilization of the carbonaceous structure at comparatively moderate temperatures. Previous studies have shown that H_3_PO_4_ impregnation can enhance char formation and influence the resistance of the carbonaceous matrix to oxidation during thermal treatment. Fierro et al. reported comparable char yields for H_3_PO_4_-impregnated lignin treated in air and nitrogen at 723 K, although oxidation became increasingly important at higher temperatures [28]. Likewise, the influence of the gaseous atmosphere on H_3_PO_4_-impregnated lignocellulosic precursors has been previously investigated, demonstrating that the final carbon structure depends on the interaction among precursor characteristics, impregnation conditions, treatment temperature, and atmosphere [29]. Nevertheless, the presence of oxygen during thermal treatment may promote partial oxidative burn-off of the carbonaceous fraction and consequently affect both solid yield and pore development. Because the present study did not include an inert-atmosphere control or a direct determination of activated-carbon yield, the specific effect of the air atmosphere on the final yield and porous structure cannot be quantitatively established and should therefore be considered a limitation of the preparation procedure.

The SEM micrograph of raw coffee husk (Figure 4a) shows a compact lignocellulosic matrix composed of dense lamellar structures with no evident pore development, which is characteristic of untreated plant fibres [30,31]. The surface contains irregular fragments and longitudinal seams associated with cellulose and hemicellulose bundles, which contribute to the mechanical rigidity of the husk.

After H_3_PO_4_ activation followed by thermal treatment, the material underwent a pronounced morphological transformation. The activated carbon (Figure 4b) exhibited fractured cell walls and a well-developed porous network, with pores of different sizes distributed throughout the carbonaceous matrix. The presence of fissures crossing the carbon domains indicates extensive structural disruption during activation [32]. This transformation confirms the effectiveness of phosphoric acid in promoting biopolymer depolymerization, dehydration, and the release of volatile components, thereby generating voids that increase surface area and improve accessibility to adsorption sites [33].

After the adsorption process (Figure 4c), partial coverage of the pore walls and a smoother surface appearance can be observed, suggesting the deposition or retention of imatinib mesylate molecules on the activated carbon surface. Although the porous structure remained visible, the apparent reduction in surface roughness and partial filling of cavities support the occurrence of adsorption within accessible pores and on external active sites.

Energy-dispersive X-ray spectroscopy (EDS) analysis (Figure 5) confirmed that the activated carbon was composed predominantly of carbon and oxygen, with contents of 75.6 and 20.9 wt%, respectively. These values are comparable to those reported for activated carbons derived from other lignocellulosic precursors [15]. The corresponding C/O ratio of approximately 4 indicates a moderately oxidized surface, consistent with the presence of phosphate, phenolic, hydroxyl, and other oxygen-containing functional groups identified by FTIR. These surface functionalities may enhance the interaction capacity of the adsorbent through hydrogen bonding, dipole–dipole interactions, and π–π interactions with the aromatic and ionizable moieties of imatinib mesylate.

Minor amounts of aluminium, potassium, and silicon were also detected, with a combined content below 3 wt%. These elements can be attributed to residual inorganic constituents naturally present in the biomass precursor or introduced during the activation process. Their low concentration indicates that the post-treatment washing procedure effectively reduced ash and metallic impurities, thereby limiting the presence of inert mineral sites that could interfere with adsorption [18,32].

Overall, the morphological and elemental analyses demonstrate that phosphoric acid activation produces a porous and chemically functionalized carbon matrix enriched in aromatic carbon domains and oxygen-containing surface groups. These features are essential for the high affinity of the material toward cytotoxic pharmaceutical compounds such as imatinib mesylate.

#### Point of Zero Charge

The point of zero charge of the activated carbon was determined to be pH 6 (Figure 6), indicating that the electrochemical behaviour of the surface depends strongly on the solution pH. At pH values below pH_PZC_, surface functional groups are predominantly protonated, resulting in a positively charged surface. Under these conditions, adsorption of anionic species may be favoured, whereas electrostatic interactions with cationic species may be less favourable. Conversely, at pH values above pH_PZC_, deprotonation of surface groups produces a negatively charged surface, which can enhance the adsorption of cationic species and reduce affinity toward anionic compounds [16].

At pH 6, corresponding approximately to the pH_PZC_, the activated carbon exhibits an overall near-neutral net surface charge. Imatinib is a polybasic molecule with reported pKa values of approximately 8.07, 3.73, 2.56, and 1.52 [34]. Therefore, at pH 6, IM is expected to exist predominantly in a protonated, positively charged form. Because the adsorbent is close to its point of zero charge under these conditions, long-range electrostatic attraction between the overall carbon surface and protonated IM is not expected to be the dominant adsorption mechanism. Instead, adsorption at pH 6 is more consistently explained by non-electrostatic interactions, particularly π–π interactions between the aromatic domains of IM and the carbonaceous surface, hydrogen bonding involving oxygenated and phosphorus-containing functional groups, and dispersion/van der Waals forces. Localized electrostatic interactions with partially deprotonated surface functionalities may still contribute, despite the approximately neutral overall surface charge. This combination of interactions provides a mechanistic explanation for the high adsorption efficiency observed at pH 6.

### 2.2. Drug Adsorption Studies

#### 2.2.1. Effect of Particle Size

The effect of activated carbon particle size on the adsorption of imatinib mesylate (IM) is summarized in Table 1. The removal efficiency increased as the particle size decreased, reaching the highest value of 96.4% for the 300 µm fractions. This behaviour is consistent with adsorption theory, as smaller particles provide a larger external surface area and shorter intraparticle diffusion pathways, thereby reducing both external film resistance and internal mass-transfer limitations [35].

However, a slight decrease in removal efficiency was observed for the 250 µm fractions, which reached 95.2%. This reduction may be attributed to particle agglomeration and partial blockage of micro-, meso-, and macropores when the adsorbent particles become excessively fine. These effects can reduce the accessibility of active sites and offset the benefit of increased surface area. Therefore, a particle size of 300 µm was selected as the optimal condition, as it provided an effective balance between accessible surface area, pore availability, and reduced agglomeration tendency.

#### 2.2.2. Effect of Adsorbent Dosage

The influence of adsorbent dosage on IM removal is shown in Figure 7. The removal efficiency increased with increasing activated carbon dosage up to 0.6 g, reaching a maximum value of 97.9%. Beyond this dosage, the removal efficiency remained nearly constant between 0.6 and 1.0 g, followed by a slight decrease at higher dosages. This trend is commonly observed in adsorption systems and can be explained by the combined influence of active-site availability, concentration-gradient effects, and particle aggregation.

At low adsorbent dosages, the increase in removal efficiency is mainly associated with the greater number of available adsorption sites, which enhances solute–adsorbent interactions. However, at higher dosages, the residual solute concentration decreases, reducing the concentration gradient across the solid–liquid interface. As a result, a fraction of the active sites remains unsaturated, leading to a decrease in the equilibrium adsorption capacity, even when the percentage removal remains high. In addition, excessive adsorbent loading can favour particle–particle interactions, resulting in agglomeration and surface shielding, which reduce the effective surface area available for adsorption [36]. Therefore, an adsorbent dosage of 0.6 g was selected as the optimum value, offering the best compromise between high removal efficiency and efficient use of the adsorbent.

#### 2.2.3. pH Study

The Solution pH is a key parameter in adsorption processes because it simultaneously affects the surface charge of the adsorbent, the speciation of the adsorbate, and the dissociation state of surface functional groups. As shown in Figure 8, IM removal was strongly dependent on pH, with the highest adsorption efficiency observed in the neutral to slightly basic range. The optimum pH was identified at approximately pH 6, which agrees with the point of zero charge of the activated carbon.

At pH 6, which is close to the pH_PZC_ of the activated carbon, the adsorbent has an approximately neutral overall surface charge, whereas IM remains predominantly protonated because the solution pH is below its principal basic pKa of approximately 8.07 [34]. Consequently, the high adsorption observed at this pH should not be attributed primarily to global electrostatic attraction. Rather, π–π interactions between the aromatic structure of IM and the carbonaceous domains, hydrogen bonding with oxygenated and phosphorus-containing surface groups, and dispersion forces are likely to provide the main contributions to adsorption. Local electrostatic interactions may additionally occur between protonated IM and negatively charged individual surface sites, such as partially deprotonated phosphate or phenolic groups, even though the net surface charge of the adsorbent is approximately zero.

Under strongly acidic conditions, increased protonation of both IM and the activated carbon surface can enhance electrostatic repulsion and competition between H^+^ and IM for oxygen-containing adsorption sites, thereby decreasing adsorption efficiency. At higher pH values, progressive deprotonation of IM and changes in the ionization state of surface functional groups modify the balance among electrostatic interactions, hydrogen bonding, and hydrophobic/π–π interactions. The observed maximum adsorption near pH 6 therefore appears to result from an optimal balance between IM speciation and the chemical state of the activated carbon surface rather than from a single electrostatic mechanism. These findings are consistent with the heterogeneous and multifunctional surface chemistry expected for H_3_PO_4_-activated carbons [16,37]. Based on adsorption efficiency and operational feasibility, pH 6 was selected for subsequent experiments.

#### 2.2.4. Effect of Contact Time

The adsorption profile as a function of contact time is shown in Figure 9. For all initial IM concentrations, the adsorption process exhibited characteristic biphasic behaviour. A rapid uptake stage occurred during the first 20–30 min, followed by a slower approach to equilibrium between approximately 60 and 100 min, depending on the initial concentration. The initial fast adsorption stage can be attributed to external film diffusion and the occupation of readily accessible adsorption sites on the external surface and larger pores. In contrast, the slower stage is associated with intraparticle diffusion into smaller pores and the gradual occupation of less accessible or lower-energy adsorption sites [38]. As expected, the equilibrium adsorption capacity increased with increasing initial IM concentration, owing to the stronger concentration gradient that enhances mass transfer from the solution to the adsorbent surface. After 60 min, changes in adsorption capacity became negligible, indicating that equilibrium was effectively reached. Therefore, a contact time of 60 min was selected as the equilibrium period for subsequent kinetic and isotherm experiments, providing a suitable balance between experimental efficiency and reliable model fitting.

### 2.3. Study of Adsorption KINETICS

The kinetic behaviour of IM adsorption onto activated carbon was evaluated by fitting the experimental data to four nonlinear kinetic models: pseudo-first-order (PFO), pseudo-second-order (PSO), Elovich, and Bangham models. The fitted curves and the corresponding kinetic parameters are presented in Figure 10 and Table 2, respectively. Among the evaluated models, the PSO model provided the best description of the adsorption process, as indicated by the lowest chi-squared error values and the good agreement between the calculated and experimental equilibrium adsorption capacities across all initial concentrations. The determination coefficients ranged from 0.946 to 0.984, confirming the superior predictive performance of this model compared with the other kinetic equations.

The PSO rate constant decreased progressively as the initial IM concentration increased, from 8.009 to 6.315 g mmol^−1^ min^−1^. This decrease may be associated with enhanced diffusional resistance and the progressive saturation of high-energy adsorption sites at higher solute concentrations. The good fit of the PSO model suggests that the adsorption rate is strongly influenced by surface interactions, including electron-sharing or electron-transfer processes between the adsorbate and the adsorbent surface [39].

The PFO model provided a reasonable fit, with R^2^ values of approximately 0.93–0.94, but showed lower predictive accuracy than the PSO model, particularly near equilibrium. This limitation suggests that a simple first-order process is insufficient to describe the entire adsorption profile, especially when mass-transfer limitations become significant [40]. The Elovich model, commonly used to describe adsorption on heterogeneous surfaces, produced lower correlation coefficients, with R^2^ values ranging from 0.837 to 0.890. Nevertheless, its parameters provide useful mechanistic information. The increase in α with increasing initial concentration indicates a higher initial adsorption rate driven by a stronger concentration gradient, whereas the decrease in β suggests a gradual reduction in the average adsorption energy as surface coverage increases [39]. The Bangham model showed the weakest overall correlation, with R^2^ values between 0.756 and 0.805. This result indicates that the model assumption of predominant pore-diffusion control does not fully describe the adsorption of IM onto phosphoric acid-activated carbon. Instead, the process appears to involve the combined contribution of external film diffusion, intraparticle diffusion, and surface interactions. Overall, the kinetic results demonstrate that the PSO model best represents the adsorption of IM onto activated carbon. These findings suggest that the adsorption process is mainly controlled by surface interactions, while diffusion resistance also contributes to the overall uptake mechanism, particularly during the later stages of adsorption.

The Weber–Morris intraparticle diffusion model was further applied to evaluate the contribution of diffusion processes to the overall adsorption mechanism. The corresponding plots and parameters are shown in Figure 11 and Table 3. The adsorption process occurred in three distinct stages, indicating the sequential involvement of external mass transfer, intraparticle diffusion, and final equilibrium.

In the first stage, corresponding to 0–20 min, adsorption capacity increased rapidly, with relatively high intraparticle diffusion rate constants and strong linearity, as reflected by R^2^ values above 0.94. This stage is associated with external film diffusion and the rapid occupation of readily accessible adsorption sites on the external surface and larger pores. The near-linear behaviour suggests efficient mass transfer toward the activated carbon surface, facilitated by the mesoporous structure of the adsorbent and the low external resistance during the initial uptake period [40].

The second stage, from 20 to 60 min, was characterized by a marked decrease in kid values and an increase in intercept C. This behaviour indicates a transition from external mass transfer to intraparticle diffusion as the main rate-limiting step. During this period, IM molecules diffuse progressively into the internal pore network, where mass transfer becomes slower because of the greater resistance imposed by the microporous domains [39]. The positive intercepts obtained for all concentrations indicate that the lines do not pass through the origin, confirming that intraparticle diffusion is not the sole rate-controlling mechanism. Instead, the adsorption process follows a mixed-control mechanism involving both film diffusion and intraparticle diffusion.

In the third stage, from 60 to 100 min, the adsorption curves reached a near-horizontal plateau and the $k_{id}$ values became very low, indicating that the system was approaching equilibrium. At this stage, $q_{t}$ changed only marginally while $t^{0.5}$ continued to increase; therefore, the linearity premise of the Weber–Morris model was no longer applicable to this equilibrium plateau. Consequently, the low R^2^ values observed in Stage 3 (0.2394–0.5745) should not be interpreted as evidence of anomalous adsorption behaviour, but rather as a consequence of the negligible variation in $q_{t}$ once the system approached saturation. Thus, Stage 3 represents the final equilibrium/saturation regime rather than a distinct diffusion-controlled step. Overall, the Weber–Morris analysis indicates that IM adsorption onto activated carbon proceeds through a multistep mechanism involving rapid external mass transfer, slower intraparticle diffusion, and final equilibrium.

### 2.4. Study of Adsorption Isotherm

The equilibrium adsorption data for imatinib mesylate (IM) onto activated carbon (AC) were fitted using the Langmuir, Freundlich, Sips, and Redlich–Peterson isotherm models. The corresponding parameters are summarized in Table 4, and the fitted isotherms are shown in Figure 12.

The Langmuir model provided a good fit to the experimental data, with determination coefficients above 0.99 at all evaluated temperatures. The maximum monolayer adsorption capacity, q_max_, increased from 0.061 mmol g^−1^ at 288 K to 0.0932 mmol g^−1^ at 308 K. This increase suggests that higher temperatures favour IM uptake, probably by enhancing molecular mobility, reducing solution viscosity, and improving access to internal adsorption sites. However, the Langmuir affinity constant, KL, decreased from 48.8322 to 39.4556 L mmol^−1^ as temperature increased, indicating a slight reduction in the binding strength of individual adsorption sites at elevated temperatures. Therefore, the Langmuir parameters suggest a trade-off between total adsorption capacity and site-specific affinity.

The Freundlich model also described the equilibrium data adequately, with R^2^ values ranging from 0.9650 to 0.9844. The Freundlich constant, KF, increased from 0.0983 to 0.2193 with increasing temperature, supporting the favourable effect of temperature on adsorption capacity. In contrast, the heterogeneity factor, *n*, decreased from 3.4225 to 2.3184 as temperature increased, indicating a reduction in adsorption intensity under higher thermal conditions [41]. Nevertheless, all *n* values were greater than 1, suggesting favourable adsorption throughout the studied temperature range.

The Redlich–Peterson model produced satisfactory fits, with R^2^ values between 0.9796 and 0.9940. The nRP parameter approached unity, particularly at 308 K, where nRP = 1.0000, indicating that the adsorption behaviour tended toward the Langmuir model under this condition [42]. Although this model captured the experimental data reasonably well, its fitting accuracy was slightly lower than that of the Sips model at the highest temperature.

Among the evaluated models, the Sips model provided the best overall fit, with R^2^ values between 0.9939 and 0.9957 and low χ^2^ values. This superior performance indicates that IM adsorption onto AC occurs on a heterogeneous surface, with behaviour that combines Freundlich-type adsorption at low concentrations and Langmuir-type monolayer adsorption at higher concentrations. The Sips maximum adsorption capacity, Q_ms_, remained relatively constant, ranging from 0.0721 to 0.0784 mmol g^−1^, whereas the Sips constant, Ks, increased markedly from 32.7156 to 54.7111 L mmol^−1^ with increasing temperature. This suggests that temperature mainly influences adsorption energetics and adsorbate–adsorbent affinity rather than substantially changing the total number of available adsorption sites. The nS values ranged from 0.7267 to 1.3714, indicating a transition from more heterogeneous adsorption behaviour at lower temperature to a more homogeneous adsorption pattern at higher temperature [43].

Comparable behaviour has been reported by Dai et al. [44] for tetracycline adsorption onto straw-derived biochar. In that study, both Langmuir and Freundlich models fitted the equilibrium data satisfactorily; however, the Sips model provided the most accurate description, reflecting the coexistence of heterogeneous adsorption at low solute concentrations and monolayer adsorption at higher concentrations. This interpretation is consistent with the mixed adsorption mechanism observed in the present work.

Overall, the isotherm analysis demonstrates that IM adsorption onto coffee husk-derived AC is favourable and temperature-dependent. The best fit obtained with the Sips model confirms the heterogeneous nature of the adsorbent surface and suggests that adsorption proceeds through a combination of pore filling, surface complexation, hydrogen bonding, electrostatic interactions, and π–π interactions involving the aromatic domains and oxygenated/phosphorylated functional groups of the activated carbon.

It should be noted that the adsorption experiments in the present study were conducted using single-solute IM solutions; therefore, the selectivity of the activated carbon toward IM in complex water matrices was not experimentally determined. Previous studies have shown that the adsorption performance of activated carbons may be affected by the presence of coexisting pharmaceuticals and other constituents commonly found in wastewater. Competitive adsorption in multicomponent systems can produce antagonistic or synergistic effects depending on both adsorbate properties and surface chemistry [45]. In secondary wastewater effluent, the removal of several pharmaceuticals by granular activated carbon has been reported to decrease relative to ultrapure water because of competition with soluble organic compounds and suspended matter [46]. Similarly, wastewater matrix effects have been shown to reduce the equilibrium adsorption capacities of pharmaceuticals on biomass-derived activated carbons as a consequence of competitive interactions and pH-dependent electrostatic effects [47]. Evidence involving imatinib is also available: Arvand and Nickdoust Masouleh investigated the simultaneous extraction of imatinib and doxorubicin using a Fe_3_O_4_/graphene oxide sorbent and evaluated the effects of salt concentration and coexisting chemotherapeutic compounds, illustrating the relevance of matrix composition when assessing IM retention from aqueous samples [48]. These effects may be particularly important for the present adsorbent because of its predominantly microporous structure, since competing organic molecules may occupy high-affinity sites or restrict access to the pore network. Consequently, the adsorption capacities reported here should be regarded as representative of single-solute conditions, and future studies using binary, multicomponent, and real wastewater matrices will be required to quantitatively establish the selectivity of coffee husk-derived activated carbon toward IM.

### 2.5. Adsorption Thermodynamics

The thermodynamic parameters presented in Table 5, together with the Van’t Hoff plot shown in Figure 13, confirm that the adsorption of IM onto AC is a spontaneous and endothermic process within the evaluated temperature range (288–308 K). The negative Gibbs free energy values (ΔG° = −34.96 to −37.93 kJ mol^−1^) indicate that the process occurs spontaneously, with increasing spontaneity at higher temperatures. The progressively more negative ΔG° values suggest that adsorption affinity improves as temperature increases, reflecting enhanced molecular mobility and access to adsorption sites.

The positive enthalpy change (ΔH° = 7.88 kJ mol^−1^) demonstrates that energy input is required for adsorption to proceed, confirming the endothermic nature of the process. This value lies within the typical range for physisorption (ΔH° < 20 kJ mol^−1^), indicating that weak physical interactions predominate [49]. However, the relatively large magnitude of ΔG°, which falls between the conventional ranges for physisorption (ΔG° ≈ −20 kJ mol^−1^) and chemisorption (ΔG° ≈ −80 to −400 kJ mol^−1^), suggests that specific intermolecular interactions, such as hydrogen bonding and π–π stacking, also contribute to the overall adsorption mechanism. These interactions are facilitated by the oxygenated and phosphorylated functional groups identified on the AC surface [50].

The positive entropy change (ΔS° = 95.72 J mol^−1^ K^−1^) indicates an increase in randomness at the solid–liquid interface during adsorption, likely due to the release of pre-adsorbed water molecules and the structural rearrangement of solvent species around the active sites [51].

Collectively, these findings confirm that the adsorption of imatinib mesylate onto phosphoric acid–activated carbon is thermodynamically favorable, driven primarily by physisorption mechanisms with minor contributions from specific surface interactions, and enhanced by the material’s surface functionality and hierarchical porosity.

### 2.6. Reuse and Desorption

The regeneration performance of the activated carbon was evaluated over six consecutive adsorption–desorption cycles using NaOH and HCl as washing agents (Figure 14). During the first reuse cycle, both regeneration protocols maintained high IM removal efficiency. However, from the second cycle onward, the NaOH-regenerated adsorbent showed superior and more stable performance than the HCl-treated material. The NaOH-regenerated AC retained approximately 90% of its initial adsorption capacity after two cycles and maintained more than 60% of its capacity in the subsequent cycles. In contrast, the HCl-treated samples exhibited a more pronounced decline in adsorption performance.

The higher stability observed after alkaline regeneration can be attributed to the preservation of deprotonated phosphate, phenolic, and other oxygen-containing functional groups, which maintain the accessibility and activity of adsorption sites. Conversely, repeated acid treatment may promote protonation and partial leaching of surface functionalities, particularly phosphorus-containing groups, as well as possible degradation of the carbon framework. These effects can progressively reduce the number and accessibility of effective adsorption sites, leading to lower reuse efficiency.

The desorption profiles showed an opposite trend. The HCl-treated AC exhibited a higher desorbed fraction, which increased progressively with reuse cycles and exceeded 40% by the sixth cycle. In contrast, NaOH washing resulted in comparatively lower desorption efficiency. This behaviour can be explained by the acid–base response of the adsorbent surface. In an acidic medium, protonation of surface functional groups weakens electrostatic interactions and hydrogen bonding between IM and the activated carbon, thereby facilitating desorption. Under alkaline conditions, deprotonation of surface groups can strengthen interactions with the cationic form of IM near neutral pH, hindering desorption [52,53].

These findings reveal a clear operational trade-off between regeneration stability and desorption efficiency. NaOH washing is more suitable when long-term adsorbent reuse and stable removal performance are prioritized, whereas HCl treatment is more effective for promoting IM desorption and possible contaminant recovery, although at the expense of faster loss of adsorption capacity. This behaviour is consistent with the porous morphology observed by SEM and the acid–base surface properties inferred from FTIR and pH_PZC_ analyses. Therefore, for practical applications, alkaline regeneration is recommended for routine reuse, while acidic treatment may be reserved for occasional deep desorption or surface reactivation.

### 2.7. Computational Analysis of Imatinib-Activated Carbon Interactions

Density functional theory (DFT) calculations were performed to provide molecular-level insight into the interactions between imatinib mesylate and the activated carbon surface. For this purpose, the imatinib molecule was divided into three main interaction regions based on its electrostatic potential distribution, as illustrated in Figure 15. This segmentation enabled the independent evaluation of the interaction between each molecular region and representative functional groups present on the activated carbon surface. A similar approach has been reported by Vologzhanina [54], who demonstrated that the different functional groups of imatinib exhibit distinct intermolecular interaction patterns. Therefore, considering multiple molecular regions is essential for a more accurate interpretation of the adsorption mechanism.

Interaction region I comprises a nitrogen-containing heterocycle with sp^2^ hybridization. This region was selected because the nitrogen atom can act as an electron-pair acceptor, enabling hydrogen bonding and electrostatic interactions with oxygenated or heteroatom-containing groups on the activated carbon surface [55]. Interaction region II corresponds to the amide moiety, in which the carbonyl group acts as an electron acceptor, whereas the N–H group can function as a hydrogen-bond donor. This dual electronic character favours dipole–dipole interactions and hydrogen bonding, making this region a potential anchoring site for imatinib adsorption [56]. Interaction region III consists of a second nitrogen-containing heterocycle with greater conformational flexibility and lower planarity. This region is less favourable for efficient π–π stacking with aromatic carbon domains, although it may still contribute to the stabilization of the adsorbate–adsorbent complex through local electrostatic or hydrogen-bonding interactions.

Geometry optimization of imatinib revealed a marked molecular polarity, with a dipole moment of 5.39 Debye. This high polarity suggests that imatinib can establish electrostatic, dipole–dipole, and hydrogen-bond interactions with functionalized carbon surfaces, particularly those enriched with oxygen-containing groups such as hydroxyl, carbonyl, and carboxyl moieties [57]. The magnitude of the dipole moment also supports the division of the molecule into different interaction regions, as it reflects the coexistence of domains with distinct electron-density distributions that may contribute differently to adsorption on heterogeneous carbon surfaces [58].

Representative models of the activated carbon surface were also optimized, as shown in Figure 16. These models included pristine carbon and hydroxyl-, carboxyl-, and carbonyl-functionalized carbon surfaces. The selection of these models was based on previous X-ray photoelectron spectroscopy studies, which identify graphitic domains and oxygenated functionalities as key contributors to adsorption on activated carbons [59]. The optimized surface models exhibited dipole moments of 0.00, 1.54, 2.39, and 4.53 Debye for pristine, hydroxyl-functionalized, carboxyl-functionalized, and carbonyl-functionalized carbon, respectively. The zero-dipole moment of the pristine carbon model is consistent with an electronically symmetric aromatic system, where π–π stacking and dispersion forces are expected to dominate interactions with imatinib [60]. In contrast, the incorporation of oxygen-containing functional groups induced surface polarization, increasing the ability of the carbon surface to participate in electrostatic interactions, dipole–dipole interactions, hydrogen bonding, and enhanced van der Waals interactions with the drug molecule [61].

The interaction energy, ΔE_int_, between imatinib and the activated carbon models was calculated using Equation (1). This approach considers the difference between the total electronic energy of the adsorbate–adsorbent complex and the sum of the electronic energies of the isolated components [62]. The optimized complexes were generated by combining each activated carbon surface model with the three predefined imatinib interaction regions.

(1) $$∆E_{int}=E_{complex}-(E_{imatinib}+E_{Carbon})$$

To ensure comparable initial conditions and avoid artificial short-range repulsion, the activated carbon models were initially positioned at a distance of 3.50 Å from each interaction site of imatinib. This separation is commonly used as a starting distance in DFT studies of adsorption on carbon-based surfaces and is consistent with non-covalent interactions such as π–π stacking, hydrogen bonding, electrostatic interactions, and dispersion forces [63]. Table 6 summarizes the calculated interaction energies for the imatinib–carbon systems, considering the three imatinib interaction regions and the four surface models. The interaction energies ranged from −59.8 to −105.3 kJ mol^−1^, indicating that the interaction strength depends strongly on both the initial molecular orientation and the type of surface functionalization. These values are consistent with strong physisorption, without evidence of covalent bond formation.

For most optimized systems, the most stable configuration involved interaction zone II of imatinib. In several cases, geometries initially placed near zones I or III relaxed toward configurations dominated by zone II, confirming the preferential role of the amide-containing region as the main anchoring site. In contrast, zone III generally produced less favourable interaction energies. This lower stability can be attributed to its lower planarity, which limits efficient π–π stacking with graphitic domains, as well as its electrostatic characteristics, which may generate less favourable interactions with the drug molecule and the carbon surface.

The hydroxyl-functionalized system initially associated with zone III is particularly informative. Although this configuration formed one hydrogen bond with imatinib, its interaction energy was only −67.7 kJ mol^−1^, which was substantially lower than those obtained for zone II configurations. This result suggests that hydrogen bonding can provide local stabilization, but that the dominant contribution to adsorption arises from π–π stacking and dispersion interactions involving the aromatic domains of the carbon surface and the more favorable molecular orientation of imatinib. From a material-design perspective, increasing the number and accessibility of graphene-like domains may therefore improve the adsorption of imatinib.

Considering only zone II interactions, the pristine carbon surface exhibited the weakest affinity, with interaction energies ranging from −81.9 to −87.5 kJ mol^−1^. These values are substantially higher than the reported interaction energy for benzene adsorption on graphene, approximately 41.8 kJ mol^−1^ [64]. This is reasonable given the larger molecular size and higher polarity of imatinib. The interaction on pristine carbon is mainly attributed to π–π stacking and dispersive van der Waals forces, consistent with theoretical and experimental studies on non-functionalized carbon surfaces.

Oxygen-containing functional groups enhanced the interaction between imatinib and the carbon surface. Figure 17 shows the most stable optimized configurations for each representative surface model. The most favorable interaction energies were −87.5, −93.1, −105.3, and −100.0 kJ mol^−1^ for pristine, hydroxyl-functionalized, carboxyl-functionalized, and carbonyl-functionalized carbon, respectively. Relative to pristine carbon, surface functionalization increased the interaction strength by approximately 6% for hydroxyl groups, 14% for carbonyl groups, and 20% for carboxyl groups.

Among the evaluated models, the carboxyl-functionalized surface showed the most negative interaction energy, indicating the most favourable interaction with imatinib. The carbonyl-functionalized surface also showed strong stabilization, whereas hydroxyl functionalization produced a more moderate enhancement. These results indicate that oxygenated groups improve adsorption not only by increasing surface polarity but also by modifying the electronic structure of neighbouring aromatic carbon domains. Carboxyl and carbonyl groups are electron-withdrawing functionalities that can reduce electron density in adjacent aromatic regions, thereby decreasing electrostatic repulsion and allowing closer and more favourable π–π stacking with imatinib [65].

Overall, the DFT results indicate that imatinib adsorption on activated carbon is governed predominantly by strong physisorption involving π–π stacking, dispersion forces, dipole–dipole interactions, and hydrogen bonding. Interaction zone II, associated with the amide region of imatinib, consistently provided the most stable configurations, confirming its role as the primary anchoring site. Surface oxygenation, particularly through carboxyl and carbonyl functionalities, reinforced the interaction by enhancing surface polarization and tuning the electronic environment of graphitic domains. These findings are consistent with the experimental adsorption results and support the key role of oxygenated functional groups and aromatic carbon structures in the high adsorption affinity of coffee husk-derived activated carbon toward imatinib mesylate.

## 3. Materials and Methods

### 3.1. Preparation of Activated Carbon from Coffee Husk

Coffee husk (CH) was used as the precursor material and was collected from Jipijapa, Manabí Province, Ecuador. The sample consisted of a random mixture of bulk residues from *Coffea arabica* and *Coffea canephora* (Robusta), provided by the local producer Café San José.

The raw material was thoroughly washed with distilled water to remove surface impurities and then oven-dried at 373 K for 3 h using a Memmert SN 30 1060 oven (Schwabach, Germany). The dried husk was ground using a high-speed multifunction comminutor (Model 100 A) to obtain particles of suitable size for chemical activation.

Chemical activation was carried out by impregnating the biomass with 40% (*w*/*w*) phosphoric acid (H_3_PO_4_; Fisher Scientific, Pittsburgh, PA, USA) at a biomass-to-acid mass ratio of 1:2 (g g^−1^). The mixture was left to impregnate at room temperature for 24 h and subsequently oven-dried at 423 K for 3 h. The H_3_PO_4_-impregnated biomass was then thermally treated in a conventional muffle furnace (Thermo Scientific, Waltham, MA, USA) at 673 K for 2 h under static air conditions. This procedure was adapted from previously reported laboratory-scale H_3_PO_4_-assisted activation protocols using conventional muffle furnaces [66].

After thermal treatment, the material was cooled to room temperature in a desiccator and washed thoroughly with hot distilled water at 338–343 K to remove residual acid. A final rinse with 0.1 mol L^−1^ NaOH solution (Thermo Scientific, USA) was performed to adjust the pH to 6.5–7.0. The resulting activated carbon was dried at 383 K for 24 h and stored in an airtight container until further use.

### 3.2. Characterization of the Adsorbent Material

The physicochemical properties of the adsorbent were evaluated by N_2_ physisorption, Fourier-transform infrared spectroscopy (FTIR), Raman spectroscopy, thermogravimetric analysis (TGA), scanning electron microscopy coupled with energy-dispersive X-ray spectroscopy (SEM–EDS), and determination of the point of zero charge (pH_PZC_).

Textural characterization was performed using a (Quantachrome Instruments Nova 4200e N_2_ physisorption analyzer, Boynton Beach, FL, USA). The specific surface area was calculated using the Brunauer–Emmett–Teller (BET) method, whereas the micropore volume was determined by the t-plot method.

Surface functional groups in raw coffee husk and activated carbon were identified using a Nicolet iS5 FTIR spectrophotometer (Thermo Fisher Scientific, Waltham, MA, USA) in the wavenumber range of 4000–550 cm^−1^. Samples were prepared as KBr pellets at a sample-to-KBr mass ratio of 1:100.

Raman spectroscopy was performed using a fully integrated confocal LabRAM HR Evolution system (HORIBA Scientific, Edison, NJ, USA). Measurements were carried out using a 532 nm excitation laser, with the laser power adjusted to 3.5% to minimize thermal effects and prevent potential structural alteration of the samples.

Thermogravimetric analysis was conducted using a TGA 55 thermogravimetric analyzer (TA Instruments, New Castle, DE, USA) controlled by TRIOS software (v 5.7.1) The sample was heated from room temperature to 973 K at a heating rate of 10 K min^−1^ under an air atmosphere. Surface morphology and elemental composition were examined using a Phenom ProX SEM system (Thermo Fisher Scientific, Eindhoven, The Netherlands). Imaging was performed at 10 kV, whereas EDS analysis was conducted at 15 kV. Before analysis, the samples were oven-dried at 353 K for 24 h and stored in a desiccator to minimize moisture interference.

The point of zero charge was determined using the pH drift method. Briefly, 100 mL of distilled water was adjusted to initial pH values ranging from 2 to 12 using 0.1 mol L HCl or NaOH. Then, 0.1 g of activated carbon was added to each flask, and the suspensions were agitated at 150 rpm for 48 h [67]. The final pH was measured using a Sper Scientific benchtop pH meter. The pH_PZC_ was identified as the intersection between the initial and final pH curves.

### 3.3. Drug Adsorption Studies

The adsorption behaviour of imatinib mesylate (IM) onto the activated carbon was evaluated through batch adsorption experiments designed to assess the effects of particle size, adsorbent dosage, solution pH, contact time, and temperature. Unless otherwise stated, experiments were conducted at 298 ± 1 K using an orbital shaker operated at 120 rpm.

In each experiment, a known mass of activated carbon was added to an aqueous IM solution of predetermined concentration and agitated for a fixed contact time. After adsorption, the suspensions were filtered using Eisco Labs filter paper with a pore size of 10 µm and a diameter of 4.9 in. Prior to UV–visible spectrophotometric analysis, the filtered samples were centrifuged to remove any residual activated-carbon fines or suspended particles that could contribute to turbidity and interfere with absorbance measurements. The residual IM concentration was then determined by UV–visible spectrophotometry, with each sample measured in triplicate to ensure the reproducibility and reliability of the analytical results.

Imatinib mesylate with 98% purity was supplied by Anhui Haikang Pharmaceutical Co., Ltd., Anqing, China. A calibration curve was prepared from a 100 mg L^−1^ stock solution by serial dilution to obtain working solutions in the concentration range of 0.002–0.20 mmol L^−1^. Quantitative analysis was performed using a UV–visible spectrophotometer (Thermo Scientific Evolution 60S, USA) equipped with quartz cuvettes. The detection wavelength was set at 255 nm.

The removal efficiency, R (%), and equilibrium adsorption capacity, q_e_, were calculated using Equations (2) and (3), respectively:

(2) $$R\%:\frac{\left(C_{i}-C_{f}\right)}{C_{i}}\times 100,$$

(3) $$q_{e}=\frac{\left(C_{i}-C_{f}\right)}{W}\times V,$$

where C_i_ is the initial concentration of Imatinib mesylate (mmol L^−1^), C_f_ is the concentration after the test (mmol L^−1^), V is the volume of solution (L) and W is the weight (g) of dry adsorbent used in experiments.

The effect of particle size was evaluated using sieved activated carbon fractions with particle sizes of 2000, 850, 605, 425, 300, and 250 µm. For each experiment, 0.5 g of adsorbent was added to 50 mL of IM solution at 0.20 mmol L^−1^ and agitated for 1 h.

Adsorbent dosage experiments were performed by varying the activated carbon mass from 0.2 to 2.0 g, in 0.2 g increments, in 100 mL of IM solution at 0.20 mmol L^−1^ under the same agitation conditions.

The effect of solution pH was investigated by adjusting the initial pH of the IM solution from 2 to 12 using 0.1 mol L^−1^ NaOH or HCl. The optimal adsorbent dosage was then added, and each suspension was stirred for 1 h before determining the residual IM concentration.

Experimental data were fitted to nonlinear mathematical models using Python version 3.11. Data visualization and graphical representations were prepared using OriginPro version 2025 and ChemDraw version 23.1.1.

### 3.4. Study of Kinetics

Kinetic experiments were conducted following the methodology described by Cavalcante et al. [32], with appropriate modifications. IM solutions of 0.20, 0.24, and 0.28 mmol L^−1^ were prepared in a volume of 100 mL and adjusted to the optimal pH determined in the pH study. The optimal adsorbent dosage obtained from the dosage experiments was used in all kinetic assays.

The suspensions were agitated under control conditions for contact times ranging from 1 to 240 min. The selected sampling times were 1, 5, 10, 15, 30, 45, 60, 75, 90, 120, 180, and 240 min. At each interval, aliquots were withdrawn, filtered, and analysed spectrophotometrically to determine the residual IM concentration.

The experimental data were fitted to several kinetic models to describe the adsorption rate and infer the possible uptake mechanism. The mathematical expressions and fitting parameters corresponding to each model are summarized in Table 7.

### 3.5. Study of Thermodynamics

Thermodynamic parameters were determined to evaluate the feasibility and nature of the adsorption process between the adsorbent and the solute at 288, 298, and 308 K. The standard Gibbs free energy change (ΔG°), enthalpy change (ΔH°), and entropy change (ΔS°) were calculated using the Van’t Hoff equations:

(9) $$∆G{}^{\circ}=-RTln(k_{L})$$

(10) $$\ln k_{c}=\frac{∆S}{R}-\frac{∆H}{RT}$$

where kL is the Langmuir equilibrium constant obtained from the adsorption isotherm model, and kc is the dimensionless equilibrium constant calculated by multiplying k_L_ by 55.5 (the molar concentration of water) and by 1000 to convert units. R is the gas constant (8314 J mol^−1^ K^−1^), and T is the absolute temperature (K). The adsorption enthalpy (ΔH°) and entropy (ΔS°) were determined from the slope and intercept, respectively, of the linear plot of ln(k_L_) vs. 1/T [19]. These parameters provide insights into the spontaneity, heat exchange, and molecular disorder associated with the adsorption process.

### 3.6. Equilibrium Study

Equilibrium adsorption experiments were conducted using imatinib mesylate (IM) solutions with initial concentrations ranging from 0.16 to 0.48 mmol L^−1^. The solutions were agitated for 4 h using the optimal activated carbon (AC) dosage at controlled temperatures of 288, 298, and 308 K to assess the influence of temperature on adsorption capacity. After reaching equilibrium, the samples were filtered, and the remaining IM concentrations were quantified by UV–Vis spectrophotometry.

The equilibrium data were analyzed by fitting non-linear adsorption models using Python (version 3.11). The Langmuir, Freundlich, Sips, and Redlich–Peterson isotherm models were applied to elucidate the adsorption behavior and identify the interactions between IM molecules and the AC surface. The corresponding model equations and fitting parameters are presented in Table 8 [68].

### 3.7. Reuse and Desorption

The desorption and reuse experiments for IM adsorbed onto activated carbon (AC) were carried out following the procedures described by Kochito [33] and Mahapatra [19], with minor adaptations. The optimal dose of adsorbent was first contacted with a 0.20 mmol L^−1^ IM stock solution under the previously established adsorption conditions. After adsorption, the residual IM concentration was determined spectrophotometrically, and the spent AC was regenerated by sequential washing with 0.01 mol L^−1^ NaOH and HCl solutions under constant stirring.

The regenerated adsorbent was then oven-dried at 343 K for 2 h before being reused in the next adsorption cycle. The efficiency of IM desorption and the stability of the adsorbent were evaluated over six consecutive cycles by measuring the IM concentration in the wash solutions and assessing the adsorption performance after each regeneration step.

### 3.8. Computational Study of the Interaction Energy Between Imatinib and Activated Carbon

A computational study was performed to investigate the interactions between imatinib mesylate and representative surface functional groups of activated carbon. All quantum chemical calculations were carried out using the ORCA software (v 6.1.0) package, whereas molecular structures and electrostatic potential maps were visualized using ChimeraX (v 1.12).

Initial molecular geometries were constructed and pre-optimized using Avogadro (v 1.102.1) with the MMFF94(s) force field. This procedure is commonly used for the preliminary optimization of organic molecules and provides energetically reasonable starting conformations before quantum mechanical calculations [69].

Subsequently, preliminary optimizations were performed for several relative orientations of imatinib and the activated carbon surface models using the GFN2-xTB method with the ALPB implicit solvent model for water. The resulting geometries were then used as starting structures for full geometry optimization at the B3LYP/def2-SVP level of theory, including Grimme’s D4 dispersion correction and the CPCM implicit solvent model for water. The inclusion of dispersion correction was considered essential because non-covalent interactions, particularly π–π stacking and van der Waals forces, are expected to play a major role in the adsorption of imatinib onto graphitic carbon domains. The selected level of theory provides a suitable balance between computational cost and structural reliability for the evaluation of adsorption-related molecular interactions [70].

For the activated carbon surface, representative molecular models were selected based on an extensive review of previous studies that characterized activated carbon surface chemistry by X-ray photoelectron spectroscopy. Reports by Yang [71], Zhilong Tang [72], Nora Samghouli [73], Hai Liu [74], and Ruchika Ojha [75] consistently indicate that activated carbon surfaces contain graphitic domains together with oxygen-containing functional groups. Based on these findings, four surface models were considered: pristine carbon (C_16_H_10_), hydroxyl-functionalized carbon (C_16_H_9_OH), carboxyl-functionalized carbon (C_16_H_9_COOH), and carbonyl-functionalized carbon (C_16_H_9_CHO). The interaction energy between imatinib and each activated carbon model was calculated according to Equation (12). This interaction does not include a correction for Basis Set Superposition Error, as we are interested in the order of the preferential groups to enhance the interaction. Because the models share a similar size and chemical nature, any BSSE contributions are expected to be nearly identical and will not affect the relative energy ordering. To evaluate the influence of molecular orientation, the imatinib molecule was positioned near different interaction regions identified from its electrostatic potential distribution. For each system, the initial separation between imatinib and the activated carbon model was set to 3.50 Å to avoid artificial short-range repulsion while allowing the formation of non-covalent interactions during geometry relaxation. The optimized complexes were subsequently analyzed in terms of interaction energy, molecular orientation, hydrogen bonding, π–π interactions, and the influence of surface functionalization.

## 4. Conclusions

Activated carbon prepared from coffee husk through H_3_PO_4_ chemical activation demonstrated high efficiency as a sustainable adsorbent for the removal of imatinib mesylate (IM) from aqueous solutions. Physicochemical characterization confirmed the successful conversion of the lignocellulosic precursor into a porous carbonaceous material with surface chemistry favourable for pharmaceutical adsorption. FTIR and SEM analyses evidenced the degradation of the original biomass structure and the formation of a porous carbon matrix enriched with oxygen-containing functional groups. Raman spectroscopy further revealed the characteristic D and G bands of activated carbon, indicating a highly defective turbostratic structure composed of small sp^2^ aromatic domains embedded within a partially amorphous matrix. The presence of D_3_ and D_4_ bands confirmed the abundance of structural defects, disordered carbon domains, and oxygenated functionalities generated during phosphoric acid activation.

Thermogravimetric analysis supported these findings by showing sequential mass-loss regions associated with the removal of physically adsorbed water, decomposition of oxygenated surface groups, and oxidation of the carbonaceous framework. The pronounced mass loss between 473 and 873 K, together with a residual ash content of approximately 18 wt%, confirmed the presence of thermally labile surface functionalities and inorganic mineral constituents inherited from the coffee husk precursor or introduced during activation.

Batch adsorption experiments identified the optimal operating conditions as a particle size of 300 µm, an adsorbent dosage of 0.6 g, and pH 6. Under these conditions, a maximum removal efficiency of 97.9% was achieved. The adsorption kinetics were best described by the pseudo-second-order model, with R^2^ values ranging from 0.946 to 0.984, indicating that IM uptake was mainly governed by surface interactions. The kinetic profile further suggested a multistep adsorption mechanism involving rapid external mass transfer, followed by slower intraparticle diffusion and final equilibrium.

Equilibrium adsorption data were best fitted by the Sips isotherm model, confirming the heterogeneous nature of the activated carbon surface and suggesting a non-uniform distribution of adsorption energies. The model also indicated a tendency toward monolayer coverage at higher IM concentrations, consistent with adsorption on chemically and texturally heterogeneous biomass-derived carbon surfaces.

Density functional theory calculations provided molecular-level support for the experimental results. Among the evaluated surface models, carboxyl-functionalized carbon exhibited the strongest interaction with imatinib, with an interaction energy of −105.3 kJ mol^−1^. This enhanced affinity was attributed to the ability of oxygenated groups, particularly carboxyl functionalities, to modulate the electronic environment of aromatic carbon domains, strengthen van der Waals interactions, and favour π–π stacking. These results confirm that IM adsorption is mainly governed by strong physisorption involving π–π interactions, dispersion forces, dipole–dipole interactions, and hydrogen bonding.

Regeneration assays showed that NaOH treatment better preserved adsorption performance over repeated cycles, whereas HCl washing promoted higher desorption efficiency but caused faster loss of adsorption capacity. This behaviour highlights an operational trade-off between long-term adsorbent stability and contaminant desorption efficiency. Therefore, alkaline regeneration appears more suitable for routine reuse, while acidic treatment may be reserved for occasional deep desorption or surface reactivation.

Overall, coffee husk-derived activated carbon is a high-performance, reusable, and environmentally sustainable adsorbent for the removal of cytotoxic pharmaceutical contaminants from water. The integration of experimental adsorption studies with computational analysis provides a comprehensive understanding of the adsorption mechanism and supports the valorization of agro-industrial waste for the development of efficient materials for water treatment applications.

## Figures and Tables

**Figure 1:**
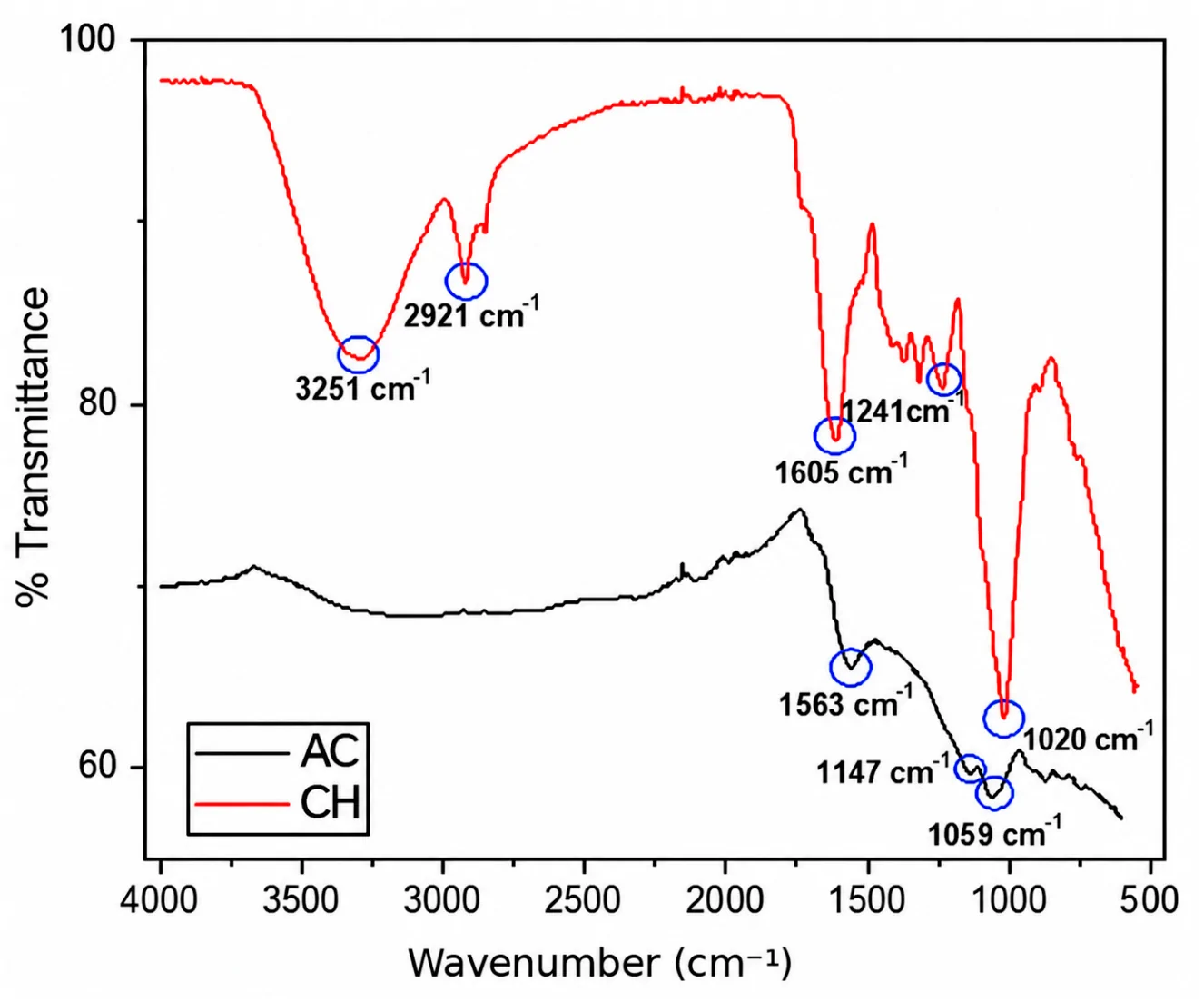
FTIR of coffee husk (CH) and activated carbon (AC).

**Figure 2:**
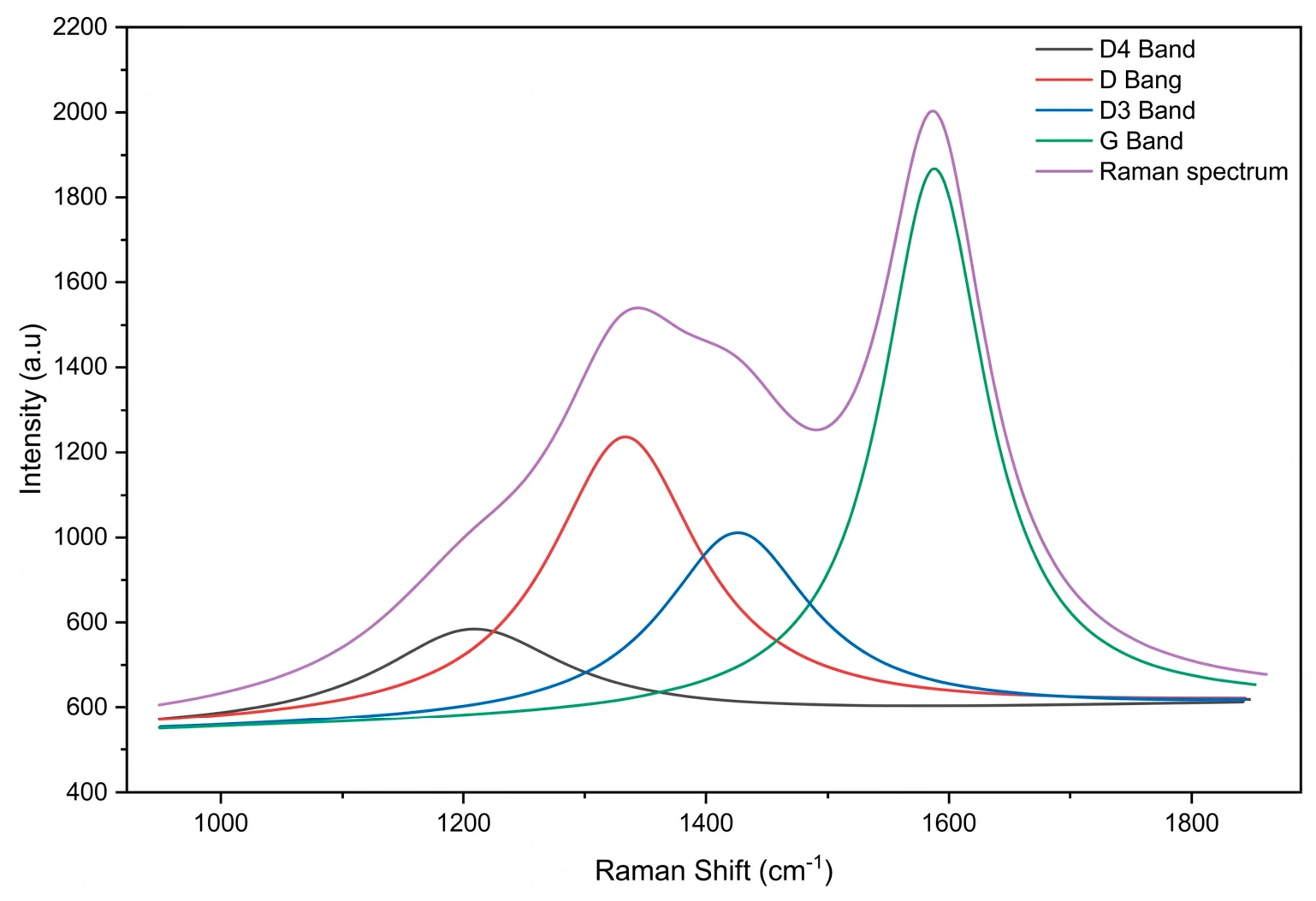
Raman Spectrum of activated carbon.

**Figure 3:**
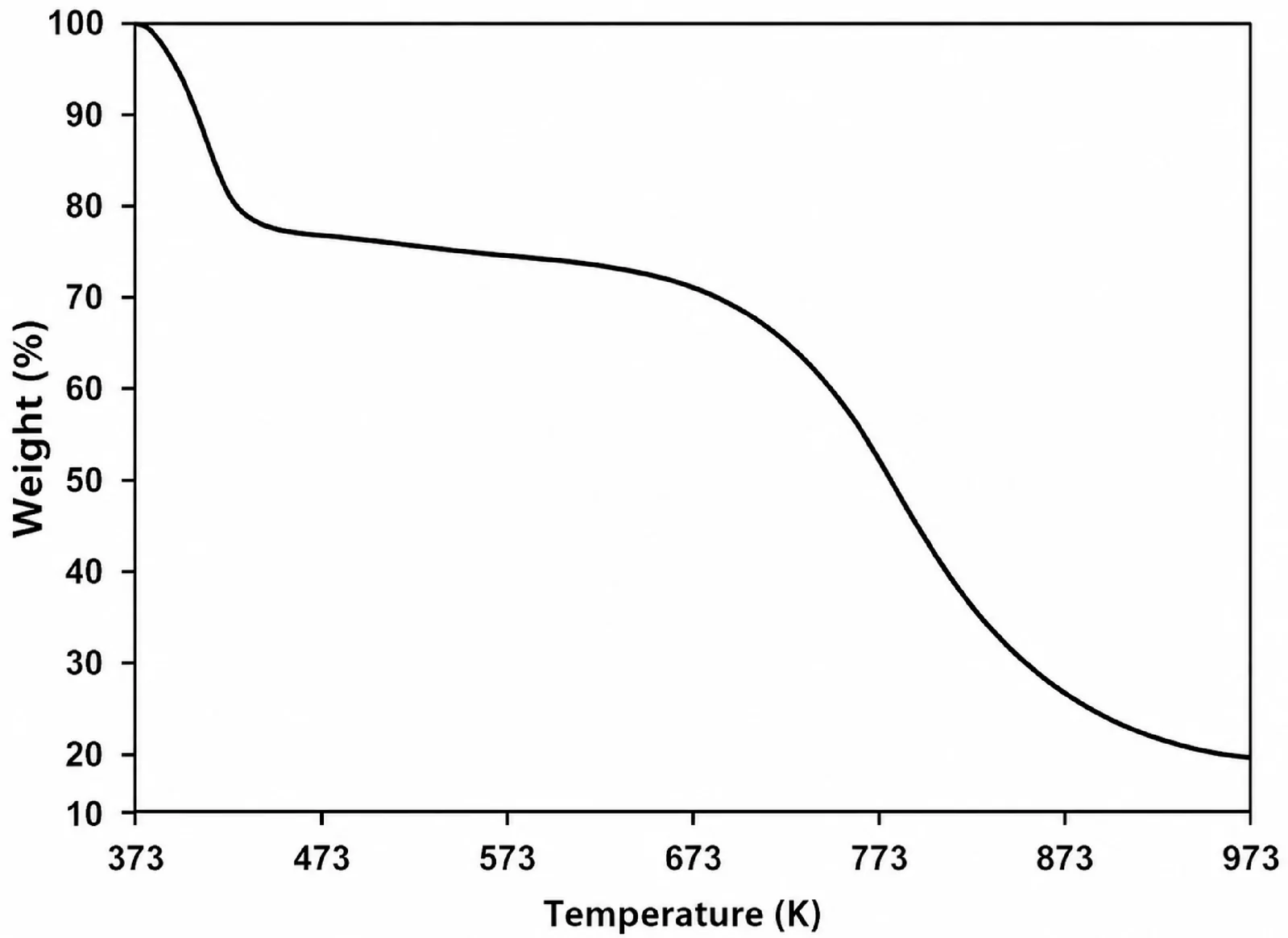
Thermogravimetric analysis of activated carbon.

**Figure 4:**
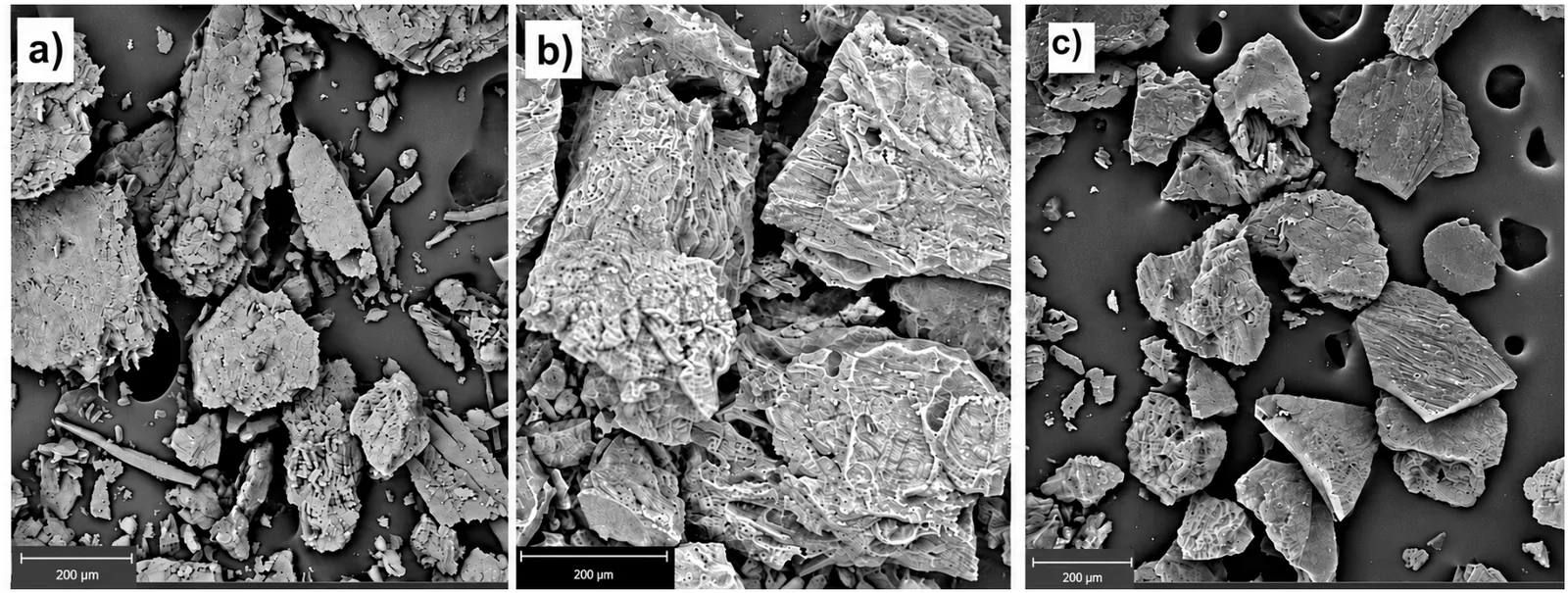
Scanning electron microscopy images of (**a**) coffee husk, (**b**) activated carbon before adsorption, and (**c**) activated carbon after adsorption.

**Figure 5:**
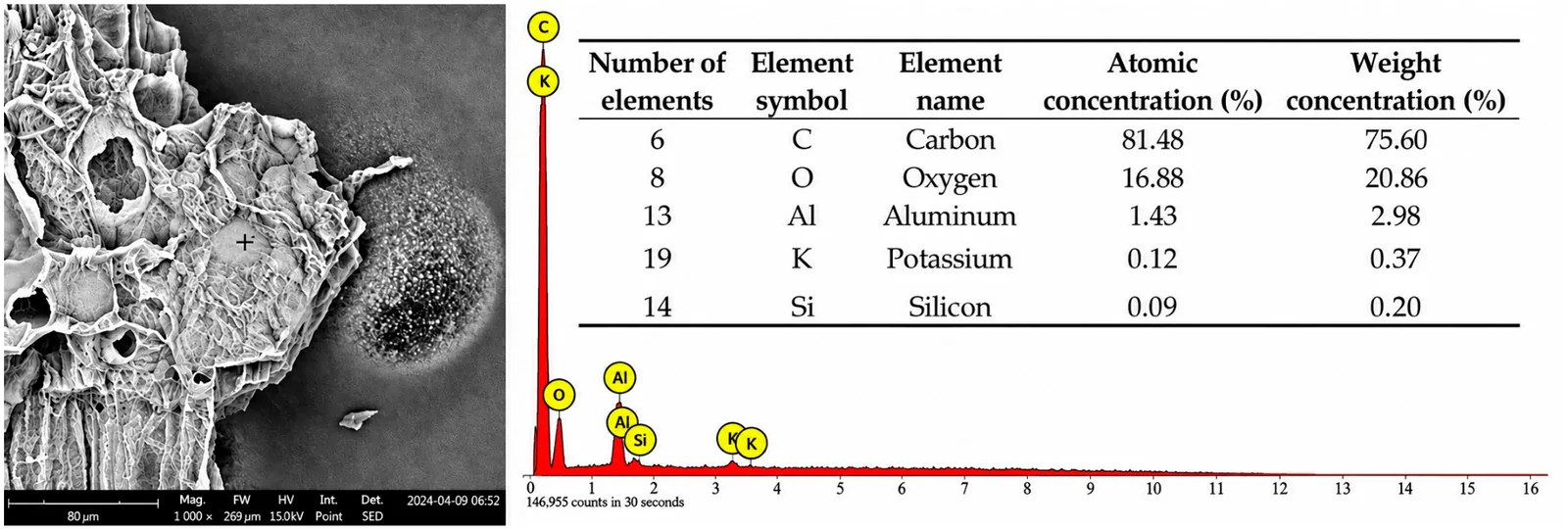
Energy-dispersive X-ray spectroscopy analysis of activated carbon prepared from coffee husk.

**Figure 6:**
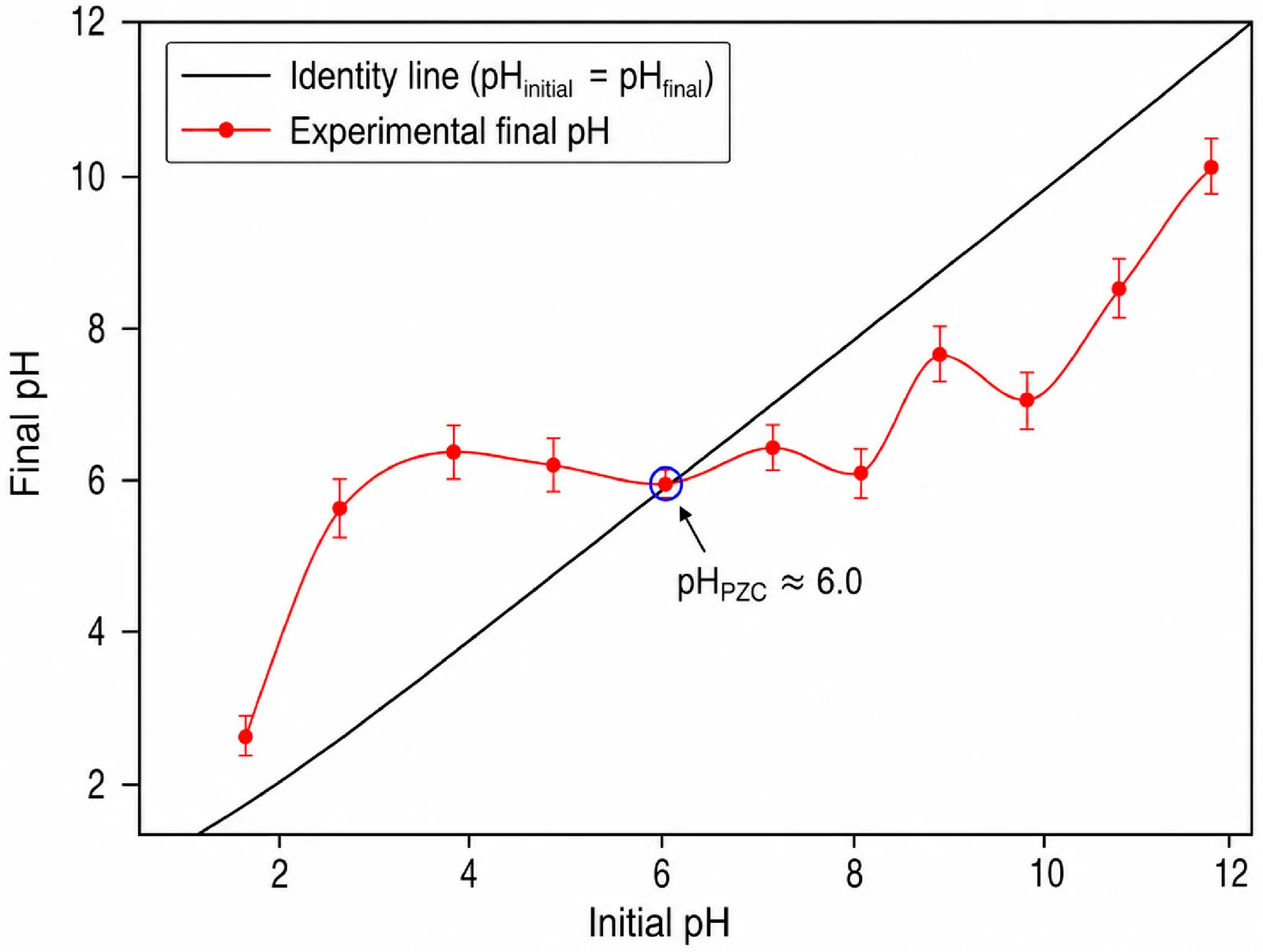
Determination of the point of zero charge (pH_PZC_) of the activated carbon. The experimental data points represent the measured final pH as a function of the initial pH, whereas the black identity line corresponds to pH_initial_ = pH_final_. The intersection of both curves indicates a pH_PZC_ of approximately 6.0.

**Figure 7:**
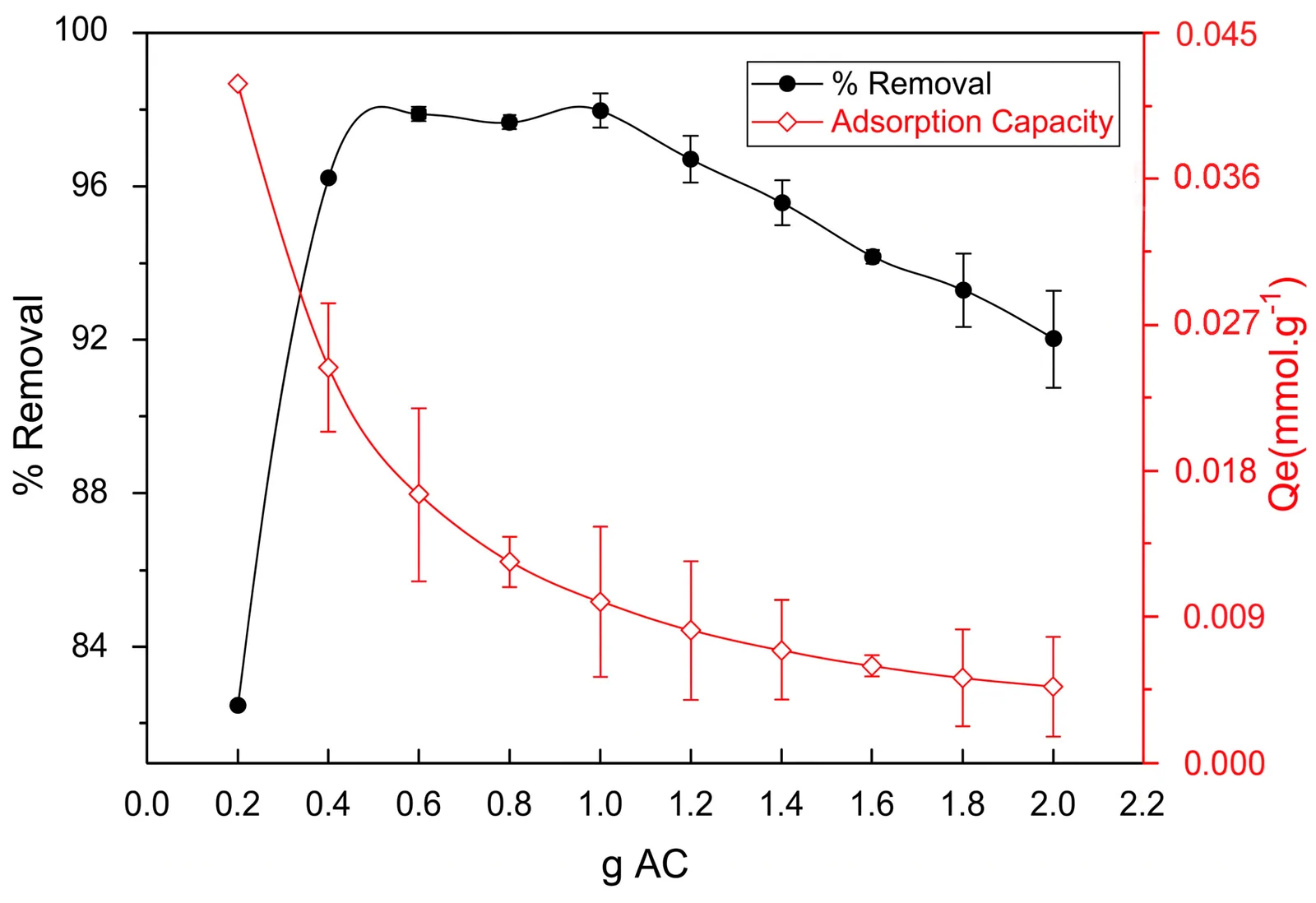
Effect of activated carbon dosage on imatinib mesylate removal and equilibrium adsorption capacity. Conditions: C_0_ = 0.20 mmol L^−1^, V = 0.10 L, particle size = 300 µm, pH = 6, T = 298 ± 1 K, 120 rpm, and t = 60 min.

**Figure 8:**
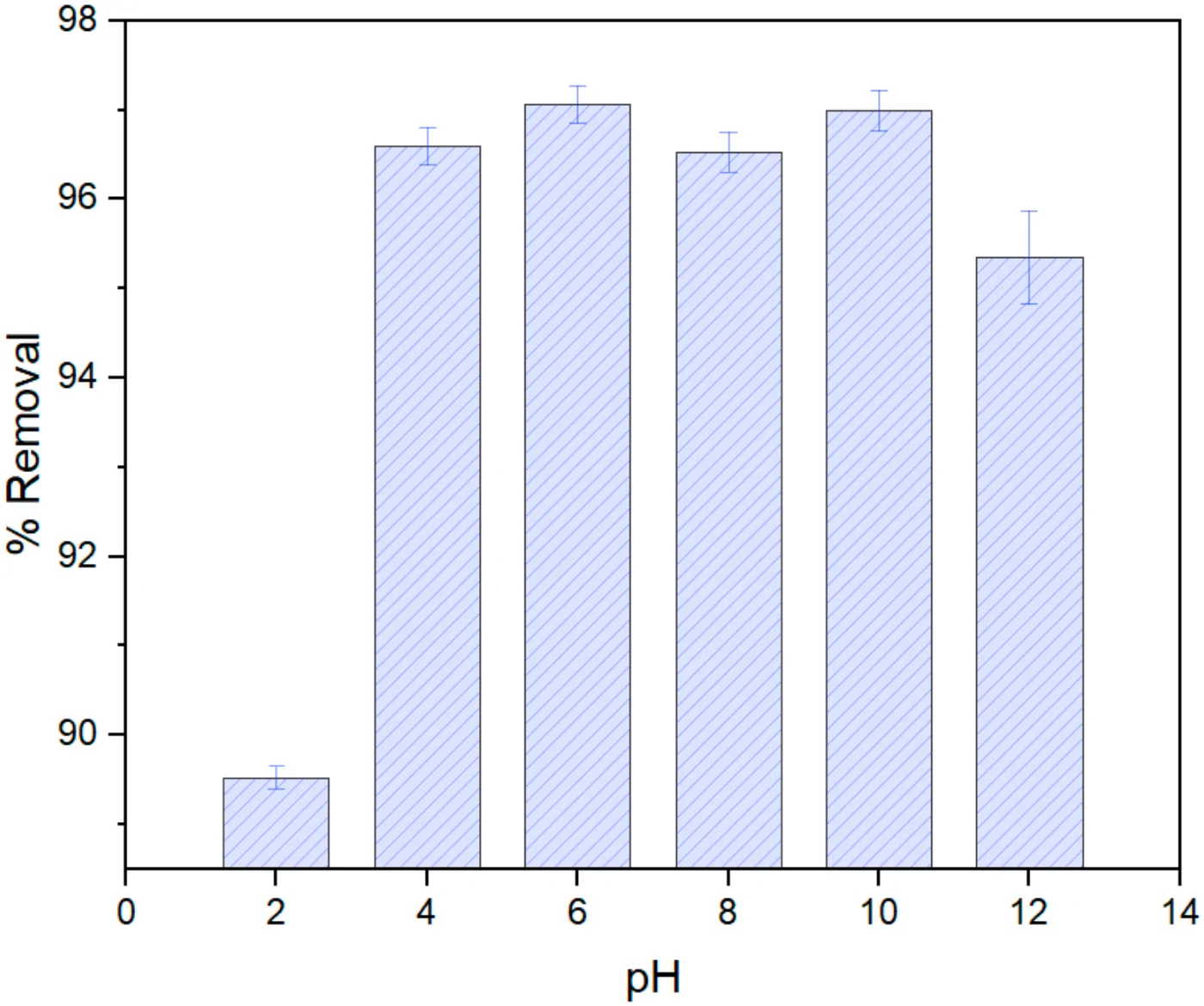
Effect of pH on the removal of imatinib mesylate by activated carbon. Conditions: C_0_ = 0.20 mmol L^−1^, m_AC_ = 0.60 g, particle size = 300 µm, V = 0.10 L, T = 298 ± 1 K, 120 rpm, and t = 60 min.

**Figure 9:**
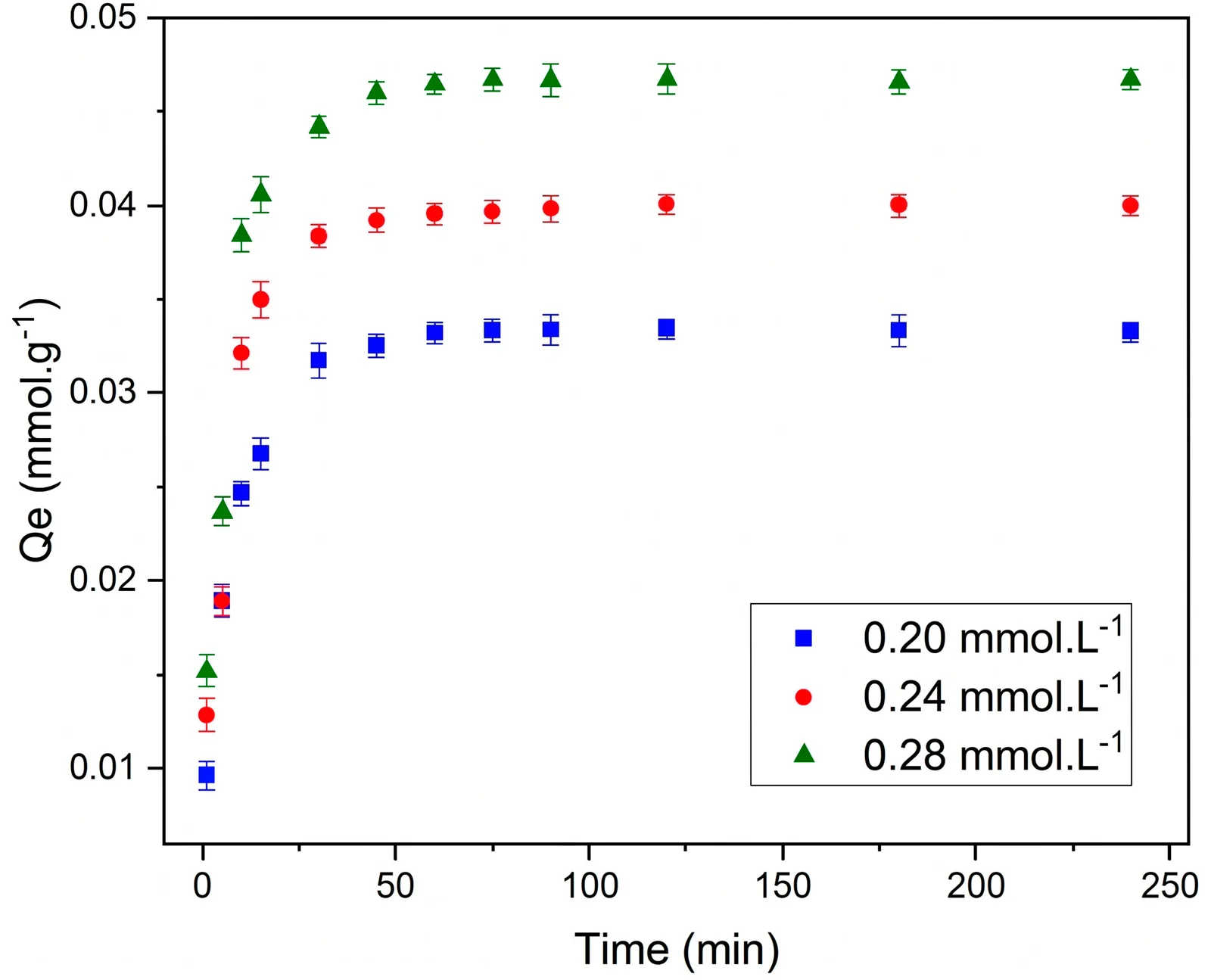
Adsorption kinetics of imatinib mesylate on activated carbon at different initial concentrations: C_0_ = 0.20, 0.24, and 0.28 mmol L^−1^. Conditions: m_AC_ = 0.60 g, particle size = 300 µm, V = 0.10 L, pH = 6, T = 298 ± 1 K, and 120 rpm.

**Figure 10:**
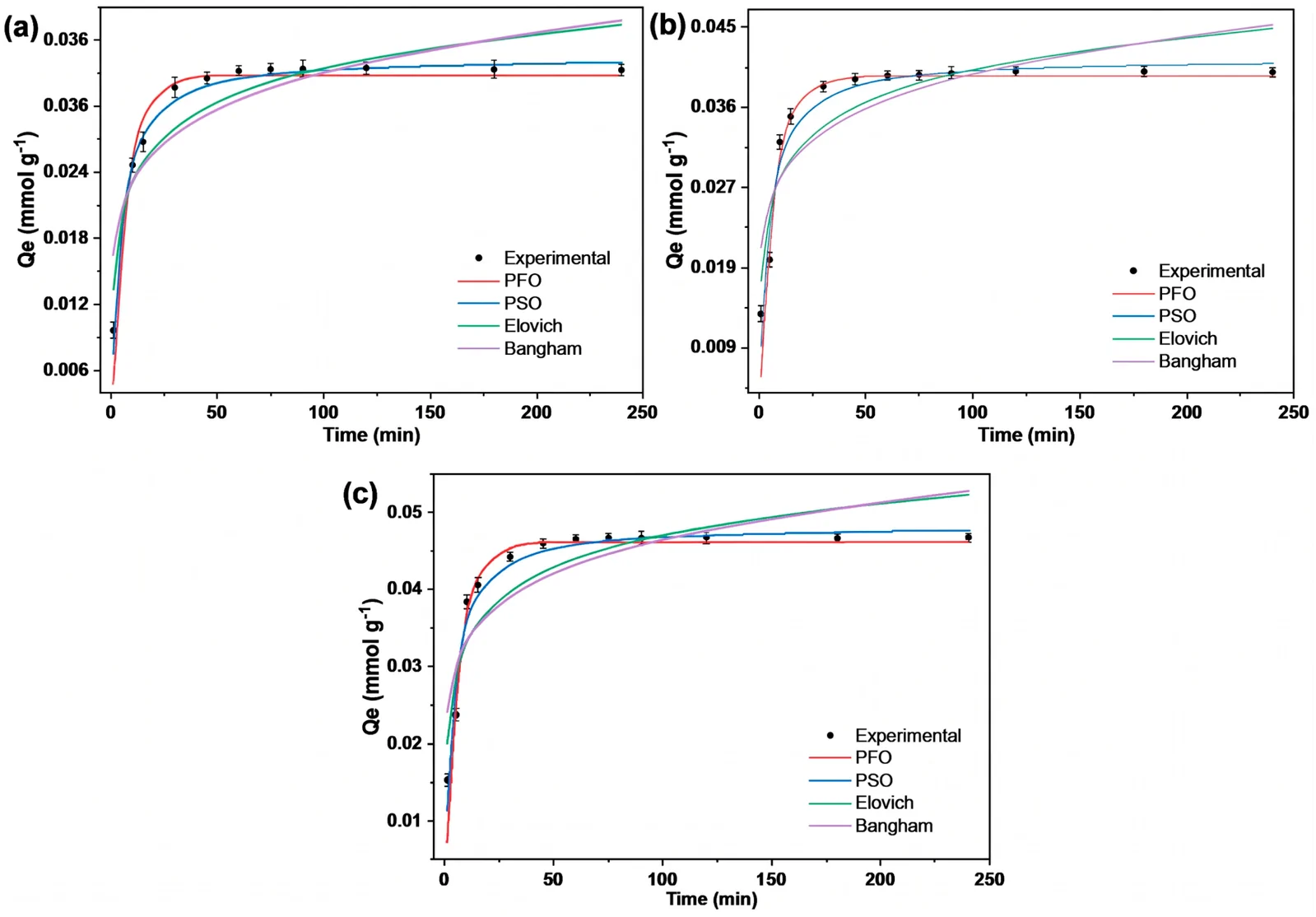
Kinetic model fitting of experimental adsorption data for imatinib mesylate onto activated carbon at different initial concentrations: (**a**) 0.20 mmol L^−1^, (**b**) 0.24 mmol L^−1^, and (**c**) 0.28 mmol L^−1^. Conditions: mAC = 0.6 g and V = 100 mL.

**Figure 11:**
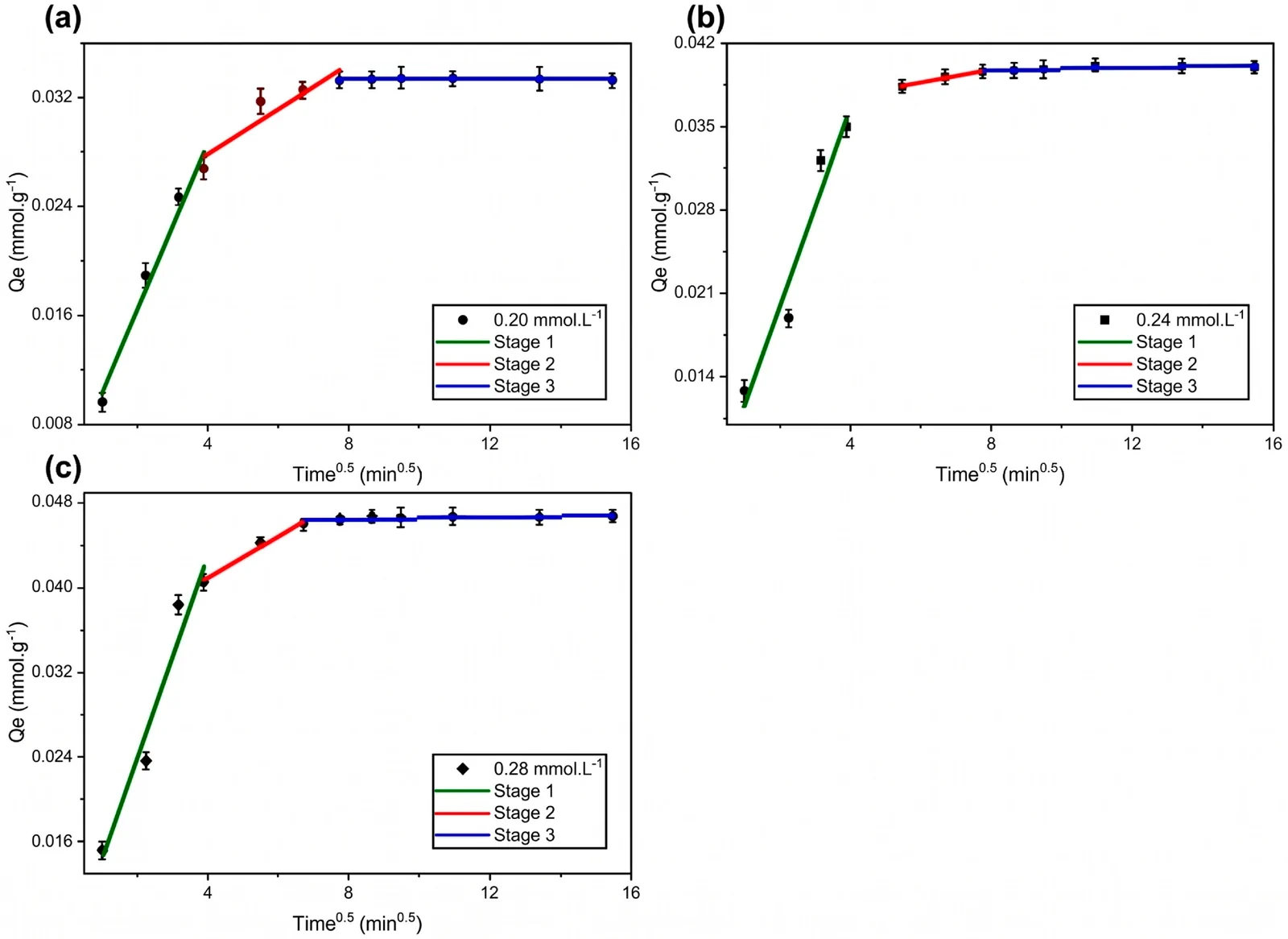
Weber–Morris intraparticle diffusion model fitted to experimental data for the adsorption of imatinib mesylate onto activated carbon at 298 K using 0.6 g of adsorbent in 100 mL of solution at pH 6. Plots correspond to different initial concentrations: (**a**) 0.20 mmol L^−1^, (**b**) 0.24 mmol L^−1^, and (**c**) 0.28 mmol L^−1^.

**Figure 12:**
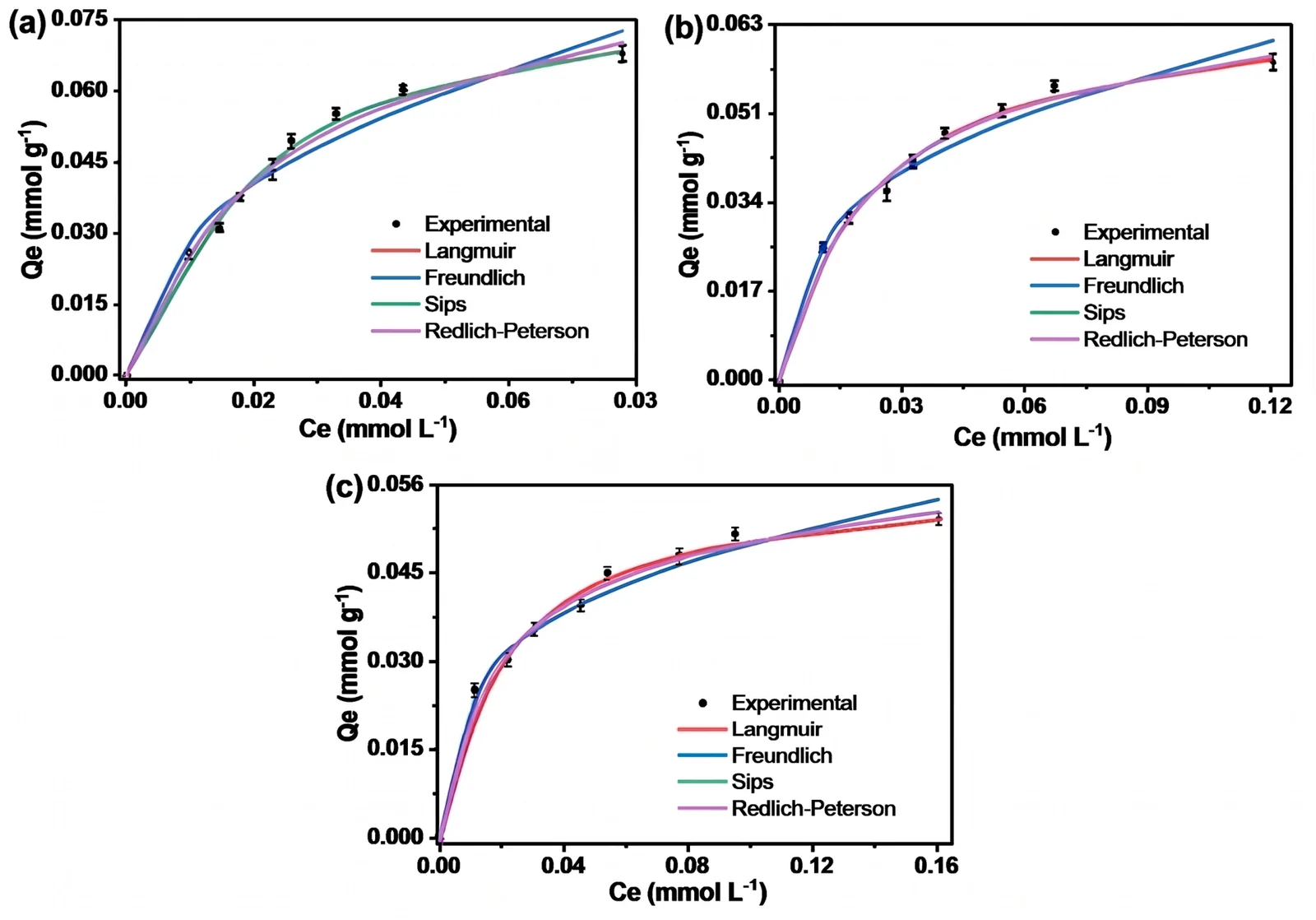
Adsorption isotherms of imatinib mesylate on activated carbon fitted to the Langmuir, Freundlich, Sips, and Redlich–Peterson models at different temperatures: (**a**) 288 K, (**b**) 298 K, and (**c**) 308 K. Experimental conditions: adsorbent mass = 0.6 g, solution volume = 100 mL, and contact time = 240 min.

**Figure 13:**
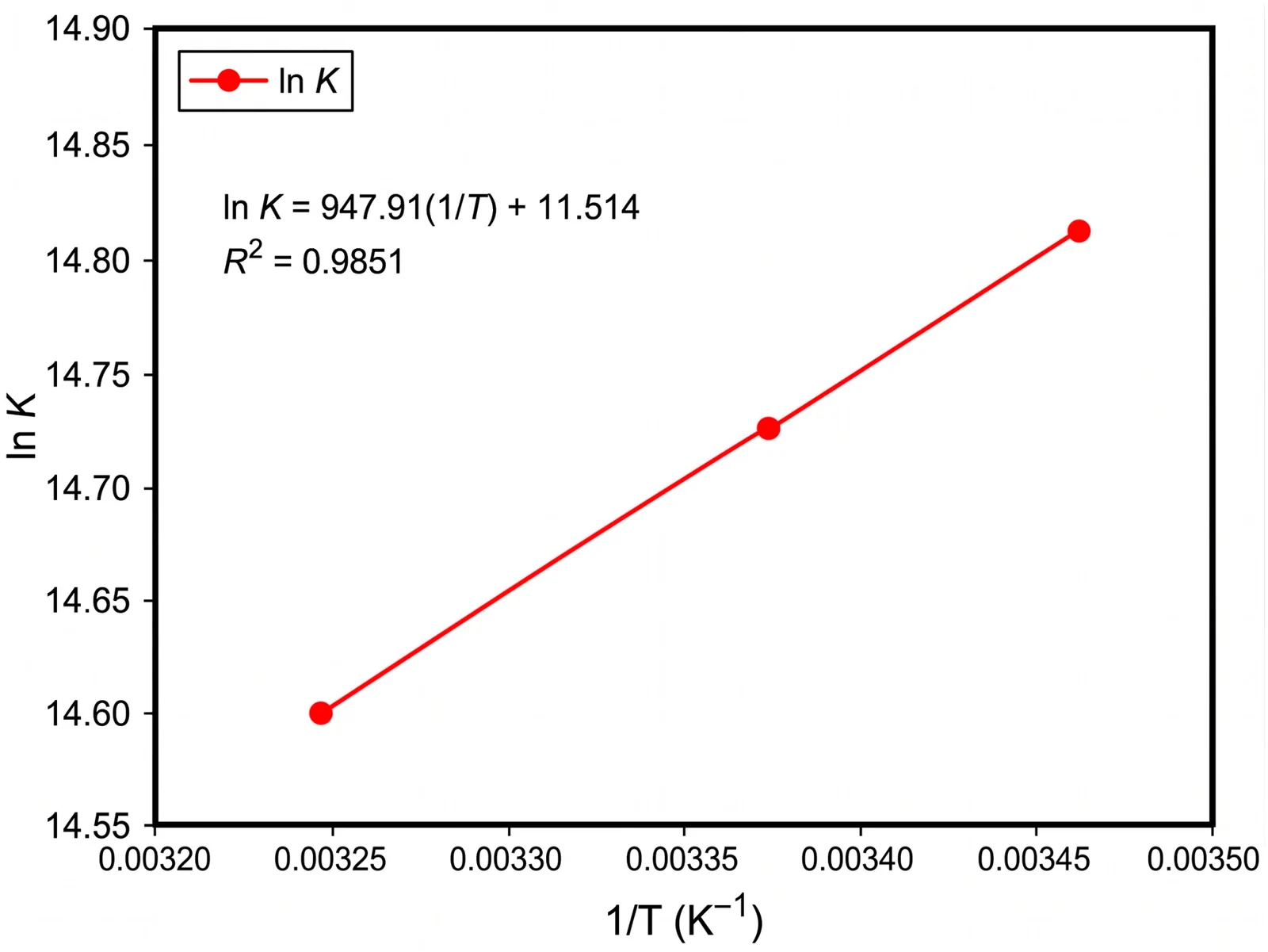
Van’t Hoff plot for the adsorption of imatinib mesylate onto coffee husk-derived activated carbon, showing the linear relationship between ln K and the reciprocal absolute temperature (1/T) used to determine the thermodynamic parameters ΔH° and ΔS°.

**Figure 14:**
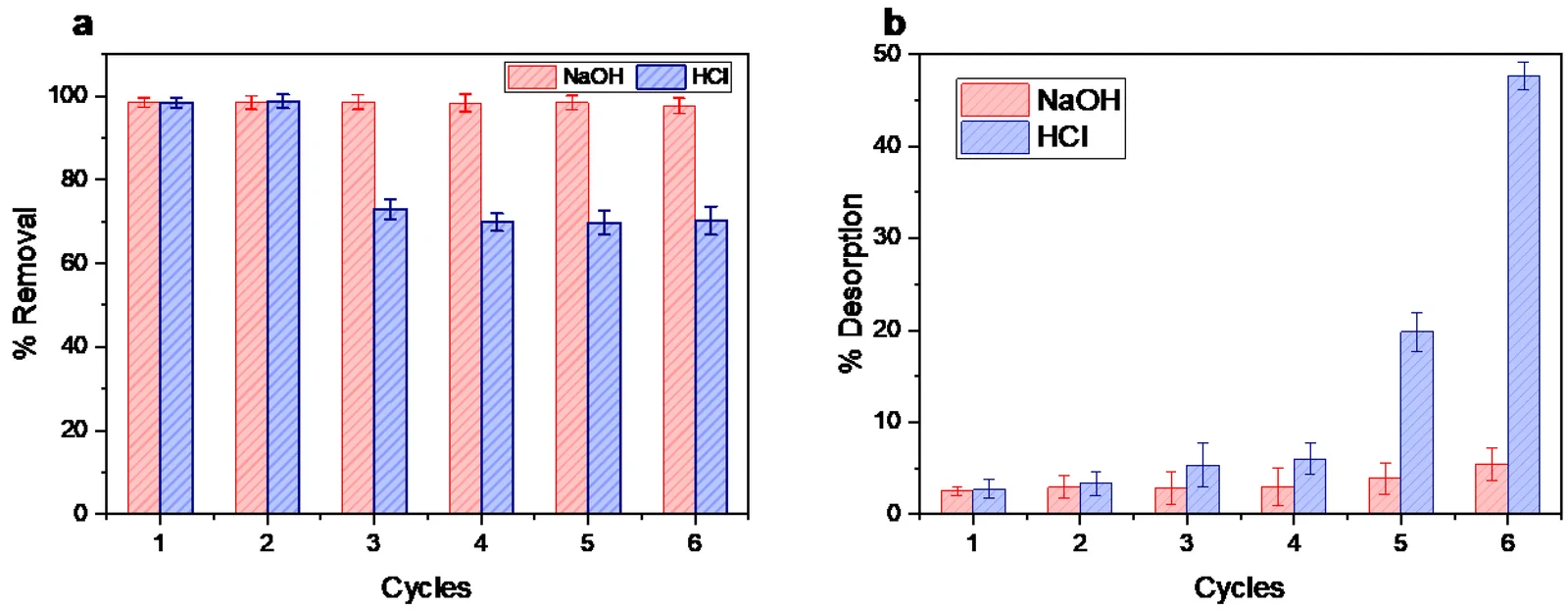
Adsorption–desorption performance of imatinib mesylate on activated carbon over six consecutive cycles using different regeneration agents: (**a**) reuse efficiency and (**b**) desorption percentage for samples washed with NaOH and HCl.

**Figure 15:**
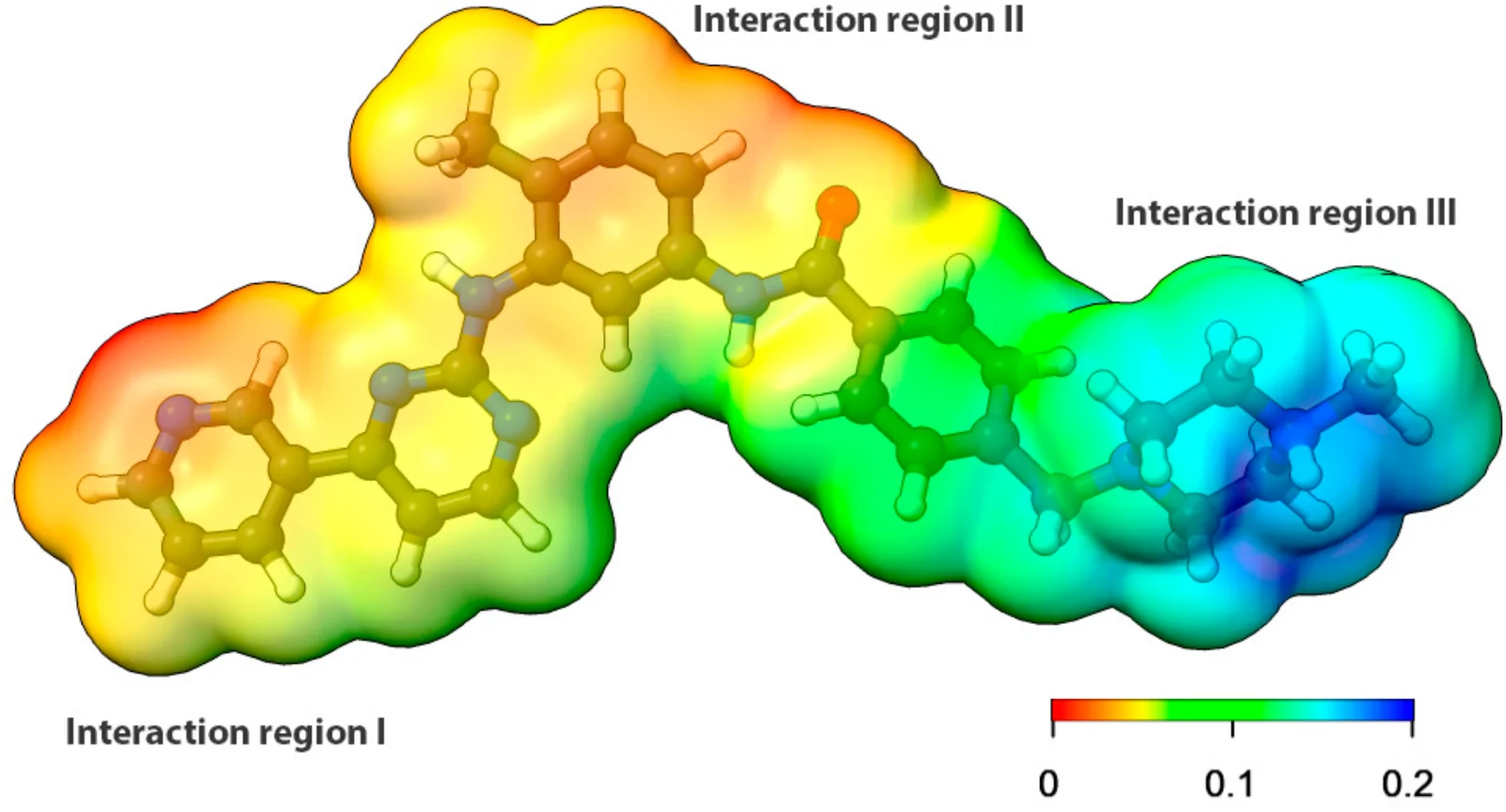
Optimized molecular structure of imatinib with electrostatic potential map highlighting the different interaction regions.

**Figure 16:**
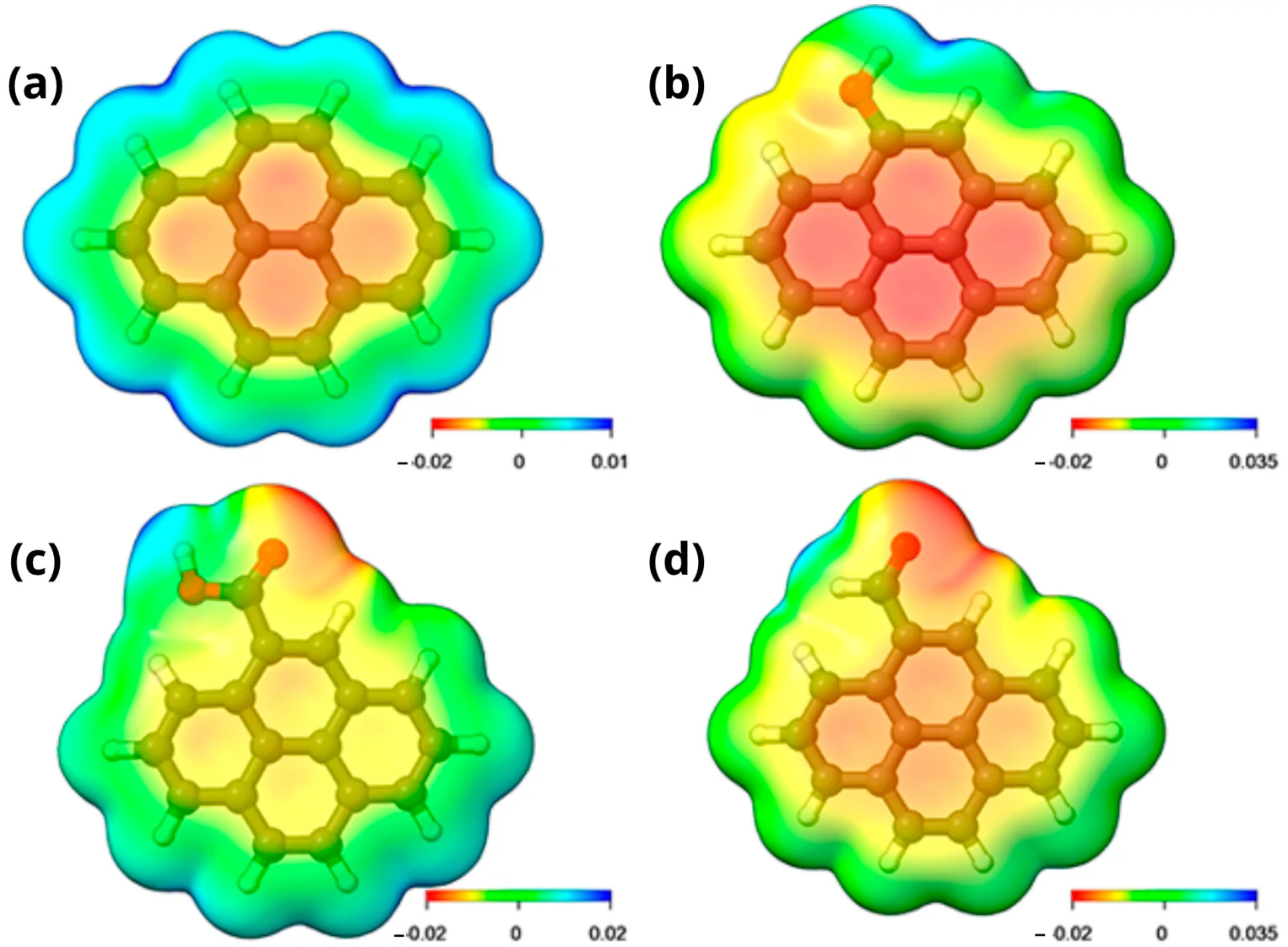
Optimized molecular structures of activated carbon surface models: (**a**) pristine carbon, (**b**) hydroxyl-functionalized carbon, (**c**) carboxyl-functionalized carbon, and (**d**) carbonyl-functionalized carbon.

**Figure 17:**
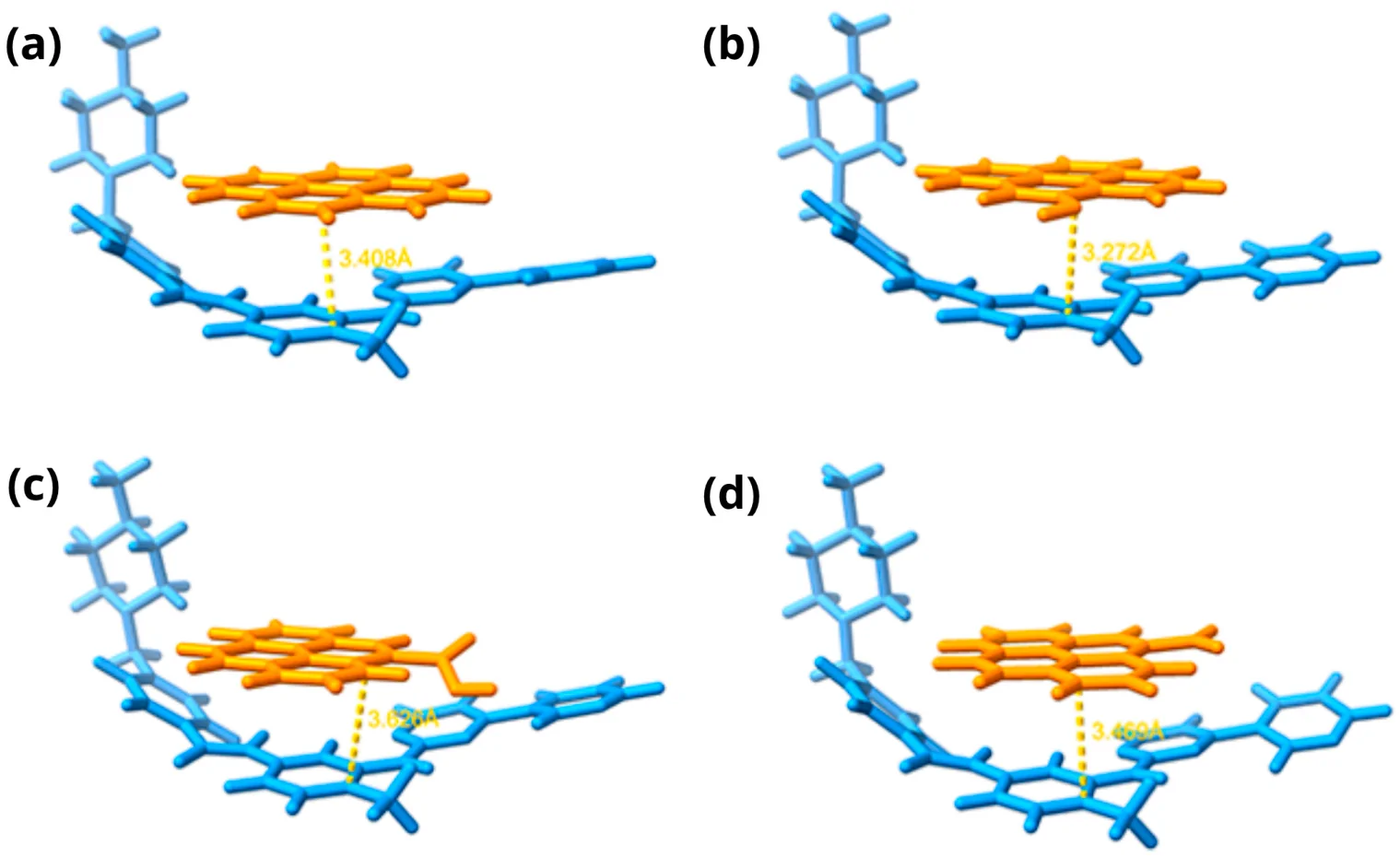
Optimized geometries of imatinib–carbon complexes showing the interaction of the drug with different surface models: (**a**) pristine carbon, (**b**) hydroxyl-functionalized carbon, (**c**) carboxyl-functionalized carbon, and (**d**) carbonyl-functionalized carbon.

**Table 1:** Effect of activated carbon particle size on the adsorption of imatinib mesylate.

Particle Size	Final Concentration (mg L^−1^)	% Removal
2.00 mm	18.03	81.9
850 µm	12.28	87.7
605 µm	7.10	92.9
425 µm	5.27	94.7
300 µm	3.55	96.4
250 µm	4.75	95.2

**Table 2:** Kinetic parameters obtained from the pseudo-first-order, pseudo-second-order, Elovich, and Bangham models for the adsorption of imatinib mesylate onto activated carbon at different initial concentrations.

Initial Concentration (mmol L^−1^)	0.20	0.24	0.28
**PPO**			
Qe, cal (mmol g^−1^)	0.0328	0.0396	0.0462
K_f_ (min^−1^)	0.1559	0.1579	0.1692
R^2^	0.9400	0.9350	0.9329
X^2^	0.0030	0.0044	0.0047
**PSO**			
Qe, cal (mmol g^−1^)	0.0345	0.0416	0.0484
k_s_ (g mmol^−1^ min^−1^)	8.0090	6.7444	6.3147
R^2^	0.9839	0.9455	0.9549
X^2^	0.0022	0.0026	0.0032
**Elovich**			
α (g mmol^−1^ min^−1^)	0.0853	0.1187	0.1661
β (g mmol^−1^)	225.6205	191.7887	168.3135
R^2^	0.8895	0.8367	0.8419
X^2^	0.0059	0.0076	0.0085
**Bangham**			
k_r_ (mmol g^−1^·min^−1/m^)	0.0165	0.0202	0.0242
A (g mmol^−1^ min^−1^)	6.6073	6.8085	7.0259
R^2^	0.8051	0.7558	0.7605
X^2^	0.0008	0.0009	0.0010

**Table 3:** Kinetic parameters obtained from the Weber–Morris intraparticle diffusion model for the adsorption of imatinib mesylate onto activated carbon at different initial concentrations.

Initial Concentration (mmol L^−1^)	0.20	0.24	0.28
**Stage 1**			
k_id_ (mmol g^−1^ min^−0.5^)	0.0061	0.0083	0.0095
C (mmol g^−1^)	0.0044	0.0035	0.0050
$R^{2}$	0.9769	0.9489	0.9550
**Stage 2**			
k_id_ (mmol g^−1^ min^−0.5^)	0.0021	0.0005	0.0019
C (mmol g^−1^)	0.0192	0.0355	0.0332
$R^{2}$	0.9052	0.9602	0.9875
**Stage 3**			
k_id_ (mmol g^−1^ min^−0.5^)	4.7 × 10^−5^	5.4 × 10^−5^	5.0 × 10^−5^
C (mmol g^−1^)	0.0327	0.0393	0.0461
$R^{2}$	0.2394	0.5745	0.3730

**Table 4:** Isotherm parameters obtained from equilibrium models at different temperatures.

Isotherm	Parameters	Temperature (K)		
		288	298	308
Langmuir	q_max_ (mmol g^−1^)	0.061	0.0736	0.0932
	k_L_ (L·mmol^−1^)	48.8322	42.7511	39.4556
	R^2^	0.9905	0.9939	0.991
	X^2^	0.00075	0.00049	0.00067
Freundlich	k_F_ [(mmol g^−1^) (L mmol^−1^)^1/n^]	0.0983	0.1368	0.2193
	n	3.4225	2.8452	2.3184
	R^2^	0.9844	0.9796	0.965
	X^2^	0.00080	0.00123	0.00268
Sips	Q_ms_ (mmol g^−1^)	0.0721	0.0784	0.0782
	k_s_ (L mmol^−1^)	32.7156	37.0915	54.7111
	n_s_	0.7267	0.8956	1.3714
	R^2^	0.9939	0.9944	0.9957
	X^2^	0.00037	0.00039	0.00043
Redlich–Peterson	K_RP_	4.2312	3.3295	3.6791
	a_RP_	57.245	42.7545	39.45
	n_RP_	0.8898	0.9707	1.0000
	R^2^	0.9928	0.9940	0.9796
	X^2^	0.00045	0.00045	0.00196

**Table 5:** Thermodynamic parameters of the adsorption process of IM.

Temperature (K)	∆G (KJ mol^−1^)	∆H (KJ mol^−1^)	∆S (J K^−1^)
288	−34.9571	7.8811	95.7250
298	−36.3696		
308	−37.9306		

**Table 6:** Interaction energies, ΔE_int_, of imatinib interacting with different activated carbon surface functional groups across the three interaction zones.

Functional Group Active Carbon	Position	Interaction Zone (After Optimization)	$∆E_{int}$ (KJ/mol)
Pristine	1	Zone II	−87.3
	2	Zone II	−81.9
	3	Zone II	−87.5
Hydroxil-Pristine	1	Zone II	−90.5
	2	Zone II	−92.1
	3	Zone II	−93.1
	4	Zone III (1 H-bond)	−67.7
Carboxyl-Pristine	1	Zone II	−102.8
	2	Zone II	−101.2
	3	Zone II	−105.3
Carbonyl-Pristine	1	Zone II	−97.7
	2	Zone II	−100.0
	3	Zone III	−59.8
	4	Zone II	−96.9

**Table 7:** Mathematical models used for the calculation of kinetic adsorption parameters.

Models	Equation	Parameter	Equation No.
Pseudo first order	$q_{t}=q_{e}\left[1-exp\left(k_{f}t\right)\right]$	$q_{t}:$ amount of adsorbate adsorbed at time t (mmol g^−1^) $q_{e}$: Adsorption capacity at equilibrium (mmol g^−1^) $k_{f}:\;$Pseudo-first-order speed constant (min^−1^) t: Contact time (min)	(4)
Pseudo second order	$q_{t}=\frac{k_{s}\;{q_{e}}^{2}t}{1+k_{s}\;q_{e}t}$ $h_{0}=k_{s}\;{q_{e}}^{2}t$	$k_{s}$: Pseudo-second-order speed constant (g mmol^−1^ min^−1^) $h_{0}$: Initial sorption rate	(5)
Intraparticle diffusion model	$q_{t}=k_{id}\;\cdot t^{0.5}+C$	C: Intraparticle diffusion constant ($mmolg^{-1}$) $k_{id}$: Constant of the rate of intraparticle diffusion (mmol g^−1^ min^−0.5^)	(6)
Elovich	$qt=\left(\frac{1}{\beta }\right)ln\left(\alpha \cdot \beta \right)+\left(\frac{1}{\beta }\right)ln\left(t\right)$	α: Initial rate of adsorption (mmol g^−1^ min^−1^) β: surface coverage and the energy of activation by chemo-separation (mmol g^−1^).	(7)
Bangham	Qt=Krt1m	$K_{r}:$ Bangham rate constant (mmol g^−1^·min^−1/m^)	(8)

**Table 8:** Mathematical models used for the calculation of adsorption equilibrium parameters.

Models	Equation	Parameter	Equation No.
Langmuir	$q_{e}=\frac{k_{L}q_{max}C_{e}}{1+k_{L}C_{e}}$	$q_{max}:$ maximum amount of adsorbate per unit weight (mmol g^−1^) $C_{e}$: Adsorption capacity at equilibrium (mmol L^−1^) $k_{L}:\;$Langmuir speed constant (L·mmol^−1^)	(11)
Freundlich	qe=kF Ce1n	$k_{F}$: Freundlich’s constant [(mmol g^−1^)(L mmol^−1^)^1/n^] n: Constant of adsorption intensity	(12)
Sips	$q_{e}=\frac{q_{ms}k_{s}{C_{e}}^{n_{s}}}{1+k_{s}{C_{e}}^{n_{s}}}$	$K_{s}:$ Constant of Sips (L·mmol^−1^) n_s_: Constant of heterogeneity	(13)
Redlich Peterson	$q_{e}=\frac{k_{RP}C_{e}}{1+a_{RP}{C_{e}}^{n_{RP}}}$	$K_{RP}:$ Constant of Redlich Peterson (L mmol^−1^) $a_{RP}$: Constant related to adsorption energy n_RP_: Deviation from the Langmuir model (L·mmol^−1^)	(14)

## Data Availability

The raw and processed data supporting the findings of this study are available from the corresponding author upon reasonable request.

## References

[1] Mishra R.K., Mentha S.S., Misra Y., Dwivedi N. (2023). Emerging pollutants of severe environmental concern in water and wastewater: A comprehensive review on current developments and future research. Water-Energy Nexus.

[2] Mišík M., Filipic M., Nersesyan A., Kundi M., Isidori M., Knasmueller S. (2019). Environmental risk assessment of widely used anticancer drugs (5-fluorouracil, cisplatin, etoposide, imatinib mesylate). Water Res..

[3] Peng B., Lloyd P., Schran H. (2005). Clinical pharmacokinetics of imatinib. Clin. Pharmacokinet..

[4] Novak M., Žegura B., Nunić J., Gajski G., Gerić M., Garaj-Vrhovac V., Filipič M. (2017). Assessment of the genotoxicity of the tyrosine kinase inhibitor imatinib mesylate in cultured fish and human cells. Mutat. Res. Genet. Toxicol. Environ. Mutagen..

[5] Leyva-Díaz J.C., Batlles-Delafuente A., Molina-Moreno V., Molina J.S., Belmonte-Ureña L.J. (2021). Removal of Pharmaceuticals from Wastewater: Analysis of the Past and Present Global Research Activities. Water.

[6] Garcia-Costa A.L., Gouveia T.I.A., Alves A., Santos M.S.F. (2023). Adsorption Technologies for the Removal of Cytostatics in Water: A Review. Water.

[7] Samal K., Mahapatra S., Hibzur Ali M. (2022). Pharmaceutical wastewater as Emerging Contaminants (EC): Treatment technologies, impact on environment and human health. Energy Nexus.

[8] Morin-Crini N., Lichtfouse E., Liu G., Balaram V., Ribeiro A.R.L., Lu Z., Stock F., Carmona E., Teixeira M.R., Picos-Corrales L.A. (2022). Worldwide cases of water pollution by emerging contaminants: A review. Environ. Chem. Lett..

[9] Ufitikirezi J.d.D.M., Filip M., Ghorbani M., Zoubek T., Olšan P., Bumbálek R., Strob M., Bartoš P., Umurungi S.N., Murindangabo Y.T. (2024). Agricultural Waste Valorization: Exploring Environmentally Friendly Approaches to Bioenergy Conversion. Sustainability.

[10] Ayalew A.A., Aragaw T.A. (2020). Utilization of treated coffee husk as low-cost bio-sorbent for adsorption of methylene blue. Adsorpt. Sci. Technol..

[11] Blachnio M., Derylo-Marczewska A., Charmas B., Zienkiewicz-Strzalka M., Bogatyrov V., Galaburda M. (2020). Activated Carbon from Agricultural Wastes for Adsorption of Organic Pollutants. Molecules.

[12] Mukherjee S., Mehta D., Dhangar K., Kumar M. (2021). Environmental fate, distribution and state-of-the-art removal of antineoplastic drugs: A comprehensive insight. Chem. Eng. J..

[13] Mustafa D., Ibrahim B., Erten A. (2024). Adsorptive removal of anticarcinogen pazopanib from aqueous solutions using activated carbon: Isotherm, kinetic and thermodynamic studies. Sci. Rep..

[14] Mbarki F., Selmi T., Kesraoui A., Seffen M. (2022). Low-cost activated carbon preparation from Corn stigmata fibers chemically activated using H3PO4, ZnCl2 and KOH: Study of methylene blue adsorption, stochastic isotherm and fractal kinetic. Ind. Crops Prod..

[15] Zahed M., Jafari D., Esfandyari M. (2022). Adsorption of formaldehyde from aqueous solution using activated carbon prepared from Hibiscus rosa-sinensis. Int. J. Environ. Anal. Chem..

[16] Ngueabouo A.M.S., Tagne R.F.T., Tchuifon D.R.T., Fotsop C.G., Tamo A.K., Anagho S.G. (2022). Strategy for optimizing the synthesis and characterization of activated carbons obtained by chemical activation of coffee husk. Mater. Adv..

[17] Paredes-Laverde M., Salamanca M., Silva-Agredo J., Manrique-Losada L., Torres-Palma R.A. (2019). Selective removal of acetaminophen in urine with activated carbons from rice (*Oryza sativa*) and coffee (*Coffea arabica*) husk: Effect of activating agent, activation temperature and analysis of physical-chemical interactions. J. Environ. Chem. Eng..

[18] Prusov A.N., Prusova S.M., Radugin M.V., Bazanov A.V. (2021). Synthesis and characterization of flax shive activated carbon. Fuller. Nanotub. Carbon Nanostruct..

[19] Mahapatra U., Chatterjee A., Das C., Manna A.K. (2021). Adsorptive removal of hexavalent chromium and methylene blue from simulated solution by activated carbon synthesized from natural rubber industry biosludge. Environ. Technol. Innov..

[20] Wang Y., Zuo S., Yang J., Yoon S.H. (2017). Evolution of Phosphorus-Containing Groups on Activated Carbons during Heat Treatment. Langmuir.

[21] Cuesta A., Dhamelincourt P., Laureyns J., Martinez-Alonso A., Tascón J.M.D. (1994). Raman Microprobe Studies on Carbon Materials. Carbon.

[22] Ferrari A.C., Robertson J. (2000). Interpretation of Raman spectra of disordered and amorphous carbon. Phys. Rev. B.

[23] Shimodaira N., Masui A. (2002). Raman spectroscopic investigations of activated carbon materials. J. Appl. Phys..

[24] Dou X., Hasa I., Saurel D., Vaalma C., Wu L., Buchholz D., Bresser D., Komaba S., Passerini S. (2019). Hard carbons for sodium-ion batteries: Structure, analysis, sustainability, and electrochemistry. Mater. Today.

[25] Baek J., Shin H.S., Chung D.C., Kim B.J. (2017). Studies on the correlation between nanostructure and pore development of polymeric precursor-based activated hard carbons: II. Transmission electron microscopy and Raman spectroscopy studies. J. Ind. Eng. Chem..

[26] Cho B., Kim D., Kim T. (2022). Exceptional Properties of Hyper-resistant Armor of a Hydrothermal Vent Crab. Res. Sq..

[27] Dittmann D., Saal L., Zietzschmann F., Mai M., Altmann K., Al-Sabbagh D., Schumann P., Ruhl A.S., Jekel M., Braun U. (2022). Characterization of activated carbons for water treatment using TGA-FTIR for analysis of oxygen-containing functional groups. Appl. Water Sci..

[28] Fierro V., Torné-Fernández V., Montané D., Celzard A. (2005). Study of the decomposition of kraft lignin impregnated with orthophosphoric acid. Thermochim. Acta.

[29] Molina-Sabio M., Caturla F., Rodriguez-Reinoso F. (1995). Influence of the atmosphere used in the carbonization of phosphoric acid impregnated peach stones. Carbone N. Y..

[30] Lafi R., Montasser I., Hafiane A. (2019). Adsorption of congo red dye from aqueous solutions by prepared activated carbon with oxygen-containing functional groups and its regeneration. Adsorpt. Sci. Technol..

[31] Tomul F., Arslan Y., Başoğlu F.T., Babuçcuoğlu Y., Tran H.N. (2019). Efficient removal of anti-inflammatory from solution by Fe-containing activated carbon: Adsorption kinetics, isotherms, and thermodynamics. J. Environ. Manag..

[32] Cavalcante L.C., de Carvalho K.Q., Bassetti F.J., Coral L.A.A. (2024). Kinetic, isothermal and thermodynamic studies of Reactive Black 5 removal using rice husk ashes and powdered activated carbon. Desalin. Water Treat..

[33] Kochito J., Gure A., Abdisa N., Beyene T.T., Femi O.E. (2024). Magnetic biochar nanocomposites of coffee husk and khat (*Catha edulis*) leftover for removal of Cr (VI) from wastewater. Curr. Res. Green Sustain. Chem..

[34] Tan K.L., Ankathil R., Gan S.H. (2011). Method development and validation for the simultaneous determination of imatinib mesylate and N-desmethyl imatinib using rapid resolution high performance liquid chromatography coupled with UV-detection. J. Chromatogr. B.

[35] Castañeda-Figueredo J.S., Torralba-Dotor A.I., Pérez-Rodríguez C.C., Moreno-Bedoya A.M., Mosquera-Vivas C.S. (2022). Removal of lead and chromium from solution by organic peels: Effect of particle size and bio-adsorbent. Heliyon.

[36] Zhang F., Liu J., Wu Y., Jin L., Wang Y., Xu Z. (2022). Study on the adsorption properties of multiple-generation hyperbranched collagen fibers towards isolan-series acid dyes. RSC Adv..

[37] Banerjee S., Chattopadhyaya M.C. (2017). Adsorption characteristics for the removal of a toxic dye, tartrazine from aqueous solutions by a low cost agricultural by-product. Arab. J. Chem..

[38] Ta H.S., Le Van K., Thi T.T.L., Ha T.H. (2020). Kinetic studies on the adsorption of phenol from aqueous solution by coffee husk activated carbon. Mediterr. J. Chem..

[39] Tan K.L., Hameed B.H. (2017). Insight into the adsorption kinetics models for the removal of contaminants from aqueous solutions. J. Taiwan Inst. Chem. Eng..

[40] Wang J., Guo X. (2020). Adsorption kinetic models: Physical meanings, applications, and solving methods. J. Hazard. Mater..

[41] Andrade C.A., Zambrano-Intriago L.A., Oliveira N.S., Vieira J.S., Quiroz-Fernández L.S., Rodríguez-Díaz J.M. (2020). Adsorption Behavior and Mechanism of Oxytetracycline on Rice Husk Ash: Kinetics, Equilibrium, and Thermodynamics of the Process. Water Air Soil Pollut..

[42] Stepova K., Sysa L., Konanets R. (2022). Nonlinear Fitting of Iron Sorption on Bentonite to Theoretical Isotherm Models. Phys. Chem. Solid State.

[43] Al-Ghouti M.A., Da’ana D.A. (2020). Guidelines for the use and interpretation of adsorption isotherm models: A review. J. Hazard. Mater..

[44] Dai H., Liu Z., Xu L., Xu G., Hu Y., Zhan P., Hu F., Peng X. (2025). Remediation of tetracycline contaminated soil and water phases by straw-derived biochar: Adsorption performance and bacterial community evolution. Ind. Crops Prod..

[45] Gęca M., Wiśniewska M., Nowicki P. (2022). Biochars and activated carbons as adsorbents of inorganic and organic compounds from multicomponent systems—A review. Adv. Colloid Interface Sci..

[46] Bo L., Gao N., Liu J., Gao B. (2016). The competitive adsorption of pharmaceuticals on granular activated carbon in secondary effluent. Desalin. Water Treat..

[47] Sousa É.M.L., Otero M., Gil M.V., Ferreira P., Esteves V.I., Calisto V. (2023). Insights into matrix and competitive effects on antibiotics removal from wastewater by activated carbon produced from brewery residues. Environ. Technol. Innov..

[48] Arvand M., Masouleh A.N. (2017). Magnetic solid-phase extraction of imatinib and doxorubicin as cytostatic drugs by Fe3O4/graphene oxide nanocomposite. J. Iran. Chem. Soc..

[49] AlOthman Z.A., Habila M.A., Ali R., Abdel Ghafar A., El-din Hassouna M.S. (2014). Valorization of two waste streams into activated carbon and studying its adsorption kinetics, equilibrium isotherms and thermodynamics for methylene blue removal. Arab. J. Chem..

[50] Zeng S., Duan S., Tang R., Li L., Liu C., Sun D. (2014). Magnetically separable Ni0.6Fe2.4O4 nanoparticles as an effective adsorbent for dye removal: Synthesis and study on the kinetic and thermodynamic behaviors for dye adsorption. Chem. Eng. J..

[51] Pereira I.C., Carvalho K., Passig F., Ferreira R., Rizzo-Domingues R., Hoppen M., Macioski G., Nagalli A., Perretto F. (2018). Thermal and thermal-acid treated sewage sludge for the removal of dye reactive Red 120: Characteristics, kinetics, isotherms, thermodynamics and response surface methodology design. J. Environ. Chem. Eng..

[52] Campbell R., Xiao B., Mangwandi C. (2024). Production of activated carbon from spent coffee grounds (SCG) for removal of hexavalent chromium from synthetic wastewater solutions. J. Environ. Manag..

[53] Ahmed M.J., Dhedan S.K. (2012). Equilibrium isotherms and kinetics modeling of methylene blue adsorption on agricultural wastes-based activated carbons. Fluid Phase Equilibria.

[54] Vologzhanina A.V., Ushakov I.E., Korlyukov A.A. (2020). Intermolecular interactions in crystal structures of imatinib-containing compounds. Int. J. Mol. Sci..

[55] Scheiner S. (2021). On the Ability of Nitrogen to Serve as an Electron Acceptor in a Pnicogen Bond. J. Phys. Chem. A.

[56] Cook D. (1958). The Donor Characteristics of the Carbonyl Group. J. Am. Chem. Soc..

[57] Manley P.W., Stiefl N., Cowan-Jacob S.W., Kaufman S., Mestan J., Wartmann M., Wiesmann M., Woodman R., Gallagher N. (2010). Structural resemblances and comparisons of the relative pharmacological properties of imatinib and nilotinib. Bioorganic Med. Chem..

[58] Aleksandrov A., Simonson T. (2010). A molecular mechanics model for imatinib and imatinib:kinase binding. J. Comput. Chem..

[59] Supong A., Sinha U.B., Sinha D. (2022). Density Functional Theory Calculations of the Effect of Oxygenated Functionals on Activated Carbon towards Cresol Adsorption. Surfaces.

[60] Spaltro A., Pila M.N., Colasurdo D.D., Grau E.N., Román G., Simonetti S., Ruiz D.L. (2021). Removal of paracetamol from aqueous solution by activated carbon and silica. Experimental and computational study. J. Contam. Hydrol..

[61] Shen F., Liu J., Zhang Z., Dong Y., Gu C. (2018). Density functional study of hydrogen sulfide adsorption mechanism on activated carbon. Fuel Process. Technol..

[62] Padak B., Brunetti M., Lewis A., Wilcox J. (2006). Mercury binding on activated carbon. Environ. Prog..

[63] Qin H., Feng C., Luan X., Yang D. (2018). First-principles investigation of adsorption behaviors of small molecules on penta-graphene. Nanoscale Res. Lett..

[64] Karton A. (2022). π–π interactions between benzene and graphene by means of large-scale DFT-D4 calculations. Chem. Phys..

[65] Zhang S., Wang L., Zhang Y., Cao F., Sun Q., Ren X., Wennersten R. (2022). Effect of hydroxyl functional groups on SO_2_ adsorption by activated carbon. J. Environ. Chem. Eng..

[66] Farrera R., Marroquín C.A., Cid M., Dávila E., García H., Méndez M.A. (2017). Producción de Carbón Activado a Escala de Laboratorio a Partir de Residuos de Café. Rev. Pakbal.

[67] Pro M.A.M., Briones A.A., Muñoz E.G., Andrade A.F., Andrade K.F. (2024). Adsorption of Brilliant blue FCF (B1) and Allura Red (R40) colorants on cocoa shell: Kinetics of the process. Rev. Colomb. Quím..

[68] Moreno-Marenco A.R., Giraldo L., Moreno-Piraján J.C. (2020). Adsorption of n-butylparaben from aqueous solution on surface of modified granular activated carbons prepared from African palm shell. Thermodynamic study of interactions. J. Environ. Chem. Eng..

[69] Tornabene F., Rajendran P., Rathinasabapathy R., Chandra Kishore S., Bellucci S. (2023). Computational-Simulation-Based Behavioral Analysis of Chemical Compounds. J. Compos. Sci..

[70] Mohr T., Aroulmoji V., Ravindran R.S., Müller M., Ranjitha S., Rajarajan G., Anbarasan P. (2015). DFT and TD-DFT study on geometries, electronic structures and electronic absorption of some metal free dye sensitizers for dye sensitized solar cells. Spectrochim. Acta Part A Mol. Biomol. Spectrosc..

[71] Yang X., Wan Y., Zheng Y., He F., Yu Z., Huang J., Wang H., Ok Y.S., Jiang Y., Gao B. (2019). Surface functional groups of carbon-based adsorbents and their roles in the removal of heavy metals from aqueous solutions: A critical review. Chem. Eng. J..

[72] Tang Z., Chen B., Huang W., Liu X., Wang X., Gong X. (2025). Predicting Adsorption Performance Based on the Properties of Activated Carbon: A Case Study of Shenqi Fuzheng System. Chemosensors.

[73] Samghouli N., Bencheikh I., Azoulay K., Jansson S., El Hajjaji S. (2025). Mechanistic and reactional activation study of carbons destined for emerging pharmaceutical pollutant adsorption. Environ. Monit. Assess..

[74] Liu H., Zhang J., Ngo H.H., Guo W., Wu H., Guo Z., Cheng C., Zhang C. (2015). Effect on physical and chemical characteristics of activated carbon on adsorption of trimethoprim: Mechanisms study. RSC Adv..

[75] Ojha R., Maniam S., Niya S.M.R., Rigoni A.S., Tran K., Tanksale A., Spencer M.J., Andrews J. (2025). Insights into the structure-property relationships of activated carbon derived from phenolic resin for electrochemical storage of green hydrogen using proton battery. J. Energy Storage.

